# Copper-Based Nanostructures as Peroxidase Mimics: Elucidating the Roles of Shape, Composition, and Chloride Ion Modulation

**DOI:** 10.1021/acsnanoscienceau.6c00146

**Published:** 2026-10-13

**Authors:** Ghazaleh Salmanian, Lihua Zhang, Mourad Benamara, Abigail Bell, Jingyi Chen

**Affiliations:** † Department of Chemistry and Biochemistry, 3341 University of Arkansas, Fayetteville, Arkansas 72701, United States; ‡ Materials Science and Engineering Graduate Program, University of Arkansas, Fayetteville, Arkansas 72701, United States; § Center for Functional Nanomaterials, 8099 Brookhaven National Laboratory, Upton, New York 11973, United States; ∥ Arkansas Nano & Bio Materials Characterization Facility, 3341 University of Arkansas, Fayetteville, Arkansas 72701, United States

**Keywords:** nanozymes, peroxidase-mimicking, chloride-accelerated, copper-based, bimetallic, copper−gold

## Abstract

Nanozymes, artificial enzymes that have emerged as promising alternatives to traditional enzymes, offer high stability, versatility, and ease of production. In this study, we investigated the peroxidase-mimicking activity of Cu-based nanostructures, including nanoparticles (CuNS) and nanowires (CuNW), as well as Cu–Au alloys and intermetallic nanoparticles. The substrate 3,3′,5,5′-tetramethylbenzidine (TMB) was used to monitor the kinetics. Our results showed that CuNW exhibited 2.1-fold higher activity toward TMB oxidation than CuNS, with a *k*_cat_ value of 10.0 × 10^–4^ s^–1^. The addition of NaCl enhanced catalytic activity, decreasing the activation energy (*E*_a_) from 15.54 to 11.92 kJ/mol for CuNS and from 14.30 to 12.83 kJ/mol for CuNW. The mechanism involves a Cu­(I)/Cu­(II) cycle, where Cu metal coupling and chloride ions facilitate the reaction. The Cu_3_Au alloy nanoparticles exhibited 1.8-fold higher catalytic activity than their intermetallic counterparts, with a *k*_cat_ value of 3.68 × 10^–4^ s^–1^ due to differences in their Cu stability. Chloride ions had a positive effect on catalytic activity, lowering *E*_a_ values from 30.45 to 16.80 kJ/mol for the alloy and from 37.92 to 32.10 kJ/mol for the intermetallic. Our findings provide new insights into the development of nonprecious metal-containing catalysts as efficient nanozymes for various applications.

## Graphical abstract

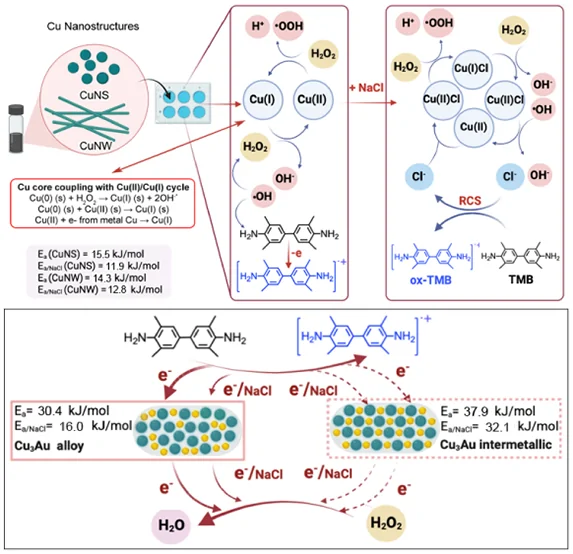

## Introduction

Nanozymes are artificial enzymes with high stability, versatility, and ease of production, making them an attractive substitute for traditional enzymes such as horseradish peroxidase (HRP) and a valuable tool in biotechnology and healthcare applications.^1–3^ HRP is widely used to detect disease biomarkers by catalyzing a reaction with a chromogenic substrate such as 3,3′,5,5′-Tetramethylbenzidine (TMB) that produces a blue-colored product, measurable at 653 nm.^4^ However, its protein-based structure makes it unstable and sensitive to harsh conditions, limiting its shelf life and usability. In contrast, nanozymes can operate under harsh conditions without degrading, do not require cold storage, and have emerged as promising alternatives. Early studies have primarily focused on utilizing metal oxides and noble metals, which catalyze reactions through Fenton-based mechanisms and electron transfer processes, respectively.^1,5^ Recent advancements have led to the design of more complex materials, including alloys, core–shell structures, and soft–hard hybrids, to create sophisticated cascade mechanisms that enhance their catalytic activity.^6^ Despite significant progress in this field, the incorporation of earth-abundant metals into nanostructures remains a challenge. To address this challenge, this study focuses on the design and development of copper-based nanostructures as nanozymes, which have the potential to serve as efficient alternatives to traditional HRP enzymes.

Copper is an attractive material for nanozyme applications due to its central role in many natural redox enzymes and its ability to switch between Cu^0^, Cu^+^, and Cu^2+^ oxidation states to facilitate electron transfer reactions.^7,8^ Cupric oxide (CuO) nanoparticles have been reported to exhibit peroxidase-like activity, enabling a colorimetric assay for H_2_O_2_ and glucose through the catalytic oxidation of TMB and phenol coupled with 4-aminoantipyrine.^9^ Similar to iron oxides, the catalytic reaction of organic substrates on CuO by H_2_O_2_ is associated with the generation of highly reactive hydroxyl radicals, a process known as Fenton chemistry.^10,11^ Terephthalic acid, acting as a radical scavenger , reacts with hydroxyl radicals to yield a fluorescent product, which can be used to quantify H_2_O_2_ in the range of 5 μM to 0.2 mM and analyze glucose and l-lactate down to 1 μM and 45 nM, respectively. The copper Fenton reaction can be accelerated by halogen ions, including chloride and bromide ions, to achieve nanomolar-range sensitivity for Cu detection.^12^ Furthermore, elemental doping of Zn into CuO (Zn-CuO) can enhance the peroxidase-mimicking activity and lower the detection limit of glucose compared to CuO alone.^13^ A composite material of CuO-Au nanoparticles has been found to generate reactive oxygen species (ROS),^14^ while Au@Cu_2_O core–shell can improve antioxidant capacity compared to pure Cu_2_O.^15^ On the other hand, Cu nanoclusters have been demonstrated as peroxidase mimetics for H_2_O_2_ detection in the range of 10 μM to 1 mM and used for glucose detection.^16^ AuCu alloy nanoparticles have also been reported with peroxidase-like properties for H_2_O_2_ and glucose detection.^17–19^ A comparison of 1:1 and 1:2 molar ratios revealed that the 1:2 alloy exhibits a lower detection limit.^17^ However, it remains unclear whether metallic Cu and its alloys undergo Fenton chemistry, follow an electron transfer mechanism similar to noble metals,^20–22^ or exhibit a combination of both. Moreover, the effects of morphological and structural properties on these reactions have yet to be fully elucidated.

In this work, we synthesize Cu with two distinct morphologies, nanoparticles and nanowires, as well as Cu alloys with Au, including Cu_3_Au alloy and intermetallic compounds, to investigate the effects of shape, composition, and chloride ions on their peroxidase-mimicking activities. Building on previous research, we consider how shapes, particularly nanowires, can enhance catalytic activities due to surface defects^23^ and how composition, including crystal structure ordering, can influence catalytic properties.^24^ We employ Michaelis–Menten enzyme kinetics to compare the catalytic performance of different Cu-based nanostructures using parameters such as maximum velocity (*V*_max_), Michaelis constant (K_m_), and turnover number (*k*_cat_). Our kinetic studies were conducted with both H_2_O_2_ and TMB substrates. By analyzing reaction rates, substrate affinities, and catalyst efficiencies, we propose distinct reaction mechanisms for Cu and its alloys as nanozymes. In addition, we examine the role of chloride ions in these mechanisms and estimate the changes in the reaction activation energy (*E*_a_). This study aims to provide valuable insights into the development of nonprecious metal-containing catalysts as efficient nanozymes.

## Results and Discussion

Spherical Cu nanoparticles (Cu nanospheres, CuNS) and Cu nanowires (CuNW) were synthesized through the reduction of precursors in organic solvents. Representative transmission electron microscopy (TEM) images of the CuNS and CuNW samples are shown in Figure 1A and B. Based on these TEM images, the average diameter of the CuNS was determined to be 23 ± 5 nm, while the average diameter and length of the CuNW were estimated to be 44 ± 19 nm and 5.2 ± 4.6 μm (Figure S1). Following a ligand exchange process with poly­(ethylene glycol) methyl ether (PEG), the morphologies of the PEGylated CuNS and CuNW remained unchanged, as confirmed by Figure S2. The crystalline structure of the resulting Cu nanostructures was verified by X-ray diffraction (XRD) patterns of the PEGylated CuNS and CuNW, as shown in Figure 1, C and D, respectively. The peaks at 2θ values of 43.3°, 50.5°, 74.1°, and 89.9° were indexed to the (111), (200), (220), and (311) planes of face-centered cubic (fcc) Cu (MP-30). A weak, broad band observed at 36.4° was assigned to the (111) plane of Cu_2_O (MP-361), indicating that the CuNS and CuNW were slightly oxidized to form a Cu_2_O layer on their surfaces. This oxide layer was further confirmed by scanning transmission electron microscopy–high-angle annular dark-field (STEM-HAADF) images and energy-dispersive X-ray spectroscopy (EDX) elemental maps (Figure 1E–F), which revealed an oxygen-rich shell surrounding the Cu nanostructures.

**Figure 1:**
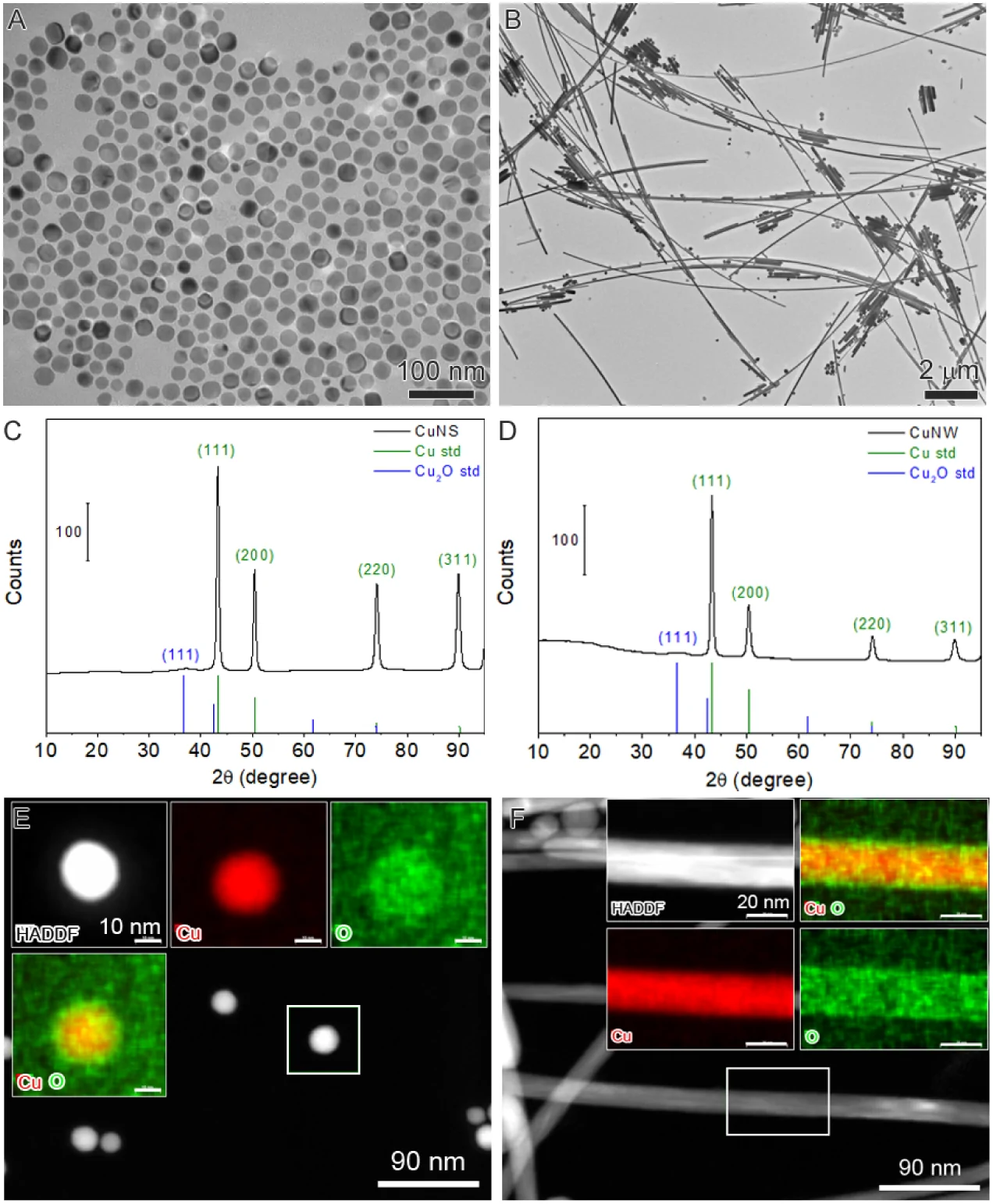
(A) TEM image of spherical Cu nanoparticles (Cu nanospheres, CuNS) with an average diameter of 23 ± 5 nm, (B) TEM image of Cu nanowires (CuNW) with an average diameter of 44 ± 19 nm and an average length of 5.2 ± 4.6 μm, (C, D) XRD pattern of PEGylated CuNS and CuNW after the ligand exchange process. The indexes in green and blue correspond to Cu (MP-30) and Cu_2_O (MP-361) standards, respectively. (E, F) STEM-HAADF images and EDX elemental maps of individual CuNS and CuNW, indicating a thin layer of O surrounding the Cu nanostructures. Cu and O are colored as red and green in the EDX maps.

The peroxidase-mimicking activities of CuNS and CuNW were investigated under steady-state conditions, with varying concentrations of TMB or H_2_O_2_ in the absence and presence of NaCl. The catalytic oxidation of TMB was monitored by measuring absorbance changes at 652 nm over time. Initial reaction rates (V_0_) were determined from the linear portions of the absorbance–time plots and calculated using the molar absorption coefficient of oxidized TMB (39,000 M^–1^ cm^–1^) and a 1 cm optical path length. The data were fitted to the Michaelis–Menten model and transformed into Lineweaver–Burk plots to extract the apparent kinetic parameters. As shown in Figure 2, the Michaelis–Menten and Lineweaver–Burk plots demonstrate typical enzyme-like saturation kinetics toward both substrates for different Cu nanostructures in buffer solutions with and without NaCl. Using hyperbolic fitting of the Michaelis–Menten plots, the apparent kinetic parameters were extracted and summarized in Table 1.

**Table 1:** Summary of Apparent Kinetic Parameters (K_m_, *V*_max_, *k*_cat_) for TMB Oxidation Catalyzed by CuNS and CuNW Nanozymes with and without NaCl^a^

		K_m_ (mM)		*V*_max_ (nM/s)		*k*_cat_ (×10^–4^ s^–1^)		*k*_cat_/K_m_ (×10^–6^ μM^–1^ s^–1^)	
Nanozyme	Substrate	No NaCl	NaCl	No NaCl	NaCl	No NaCl	NaCl	No NaCl	NaCl
CuNS	TMB	0.37	0.55	74.16	173.00	4.70	10.96	1.34	1.99
	H_2_O_2_	120.20	81.80	181.50	248.30	11.50	15.73	0.01	0.02
CuNW	TMB	1.52	1.21	157.60	309.66	10.00	19.62	0.65	1.62
	H_2_O_2_	107.43	48.60	150.80	193.66	9.55	12.27	0.01	0.03
Cu_2_O/CuNP	TMB	0.77	-	163.05	-	10.36	-	1.36	-

a *k*_cat_ was calculated based on the Cu atom concentration (1.58 × 10^–4^ M).

**Figure 2:**
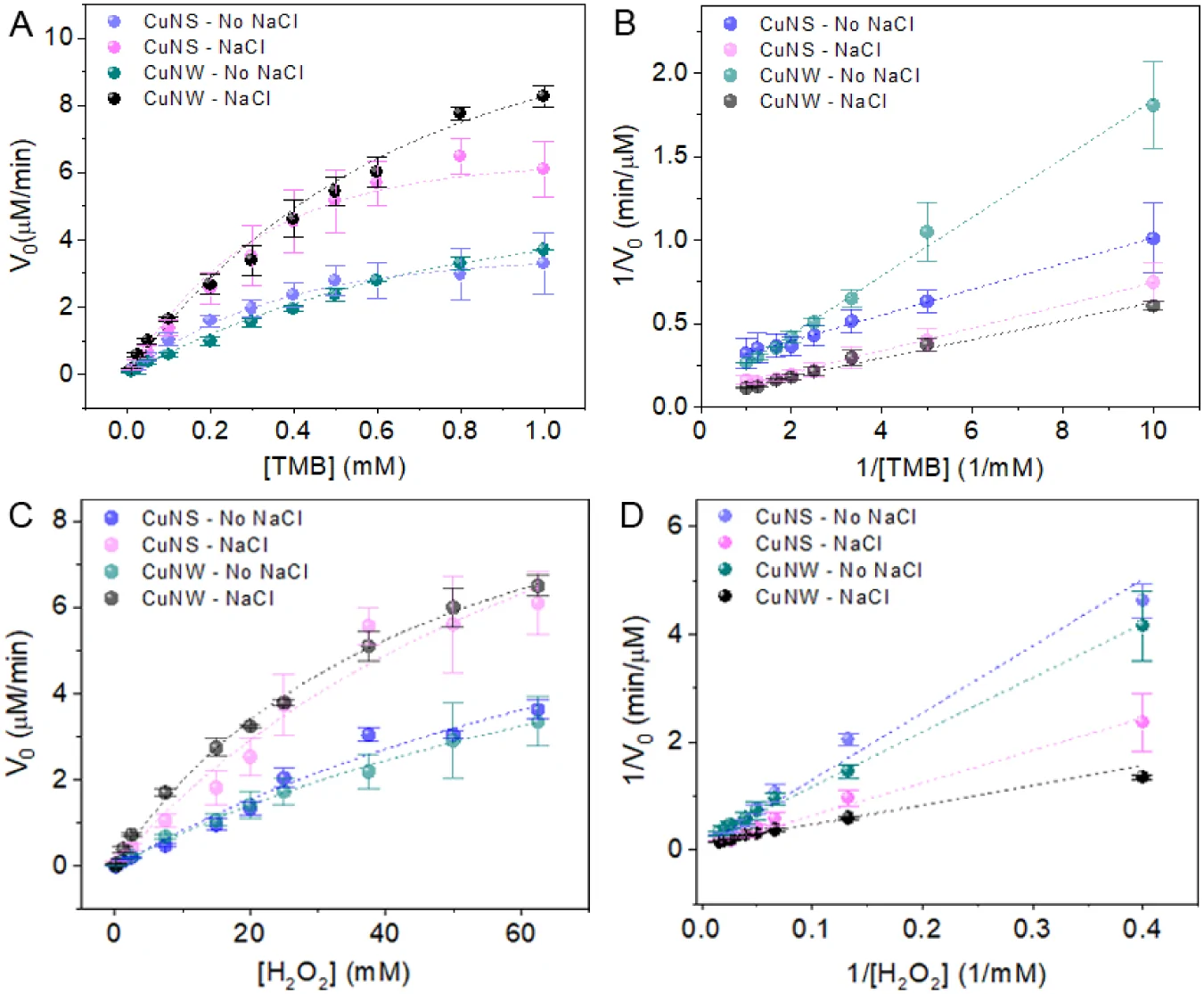
Steady-state kinetic analysis for TMB oxidation catalyzed by CuNS and CuNW nanozymes in the presence and absence of NaCl: (A) Michaelis–Menten plot of initial reaction velocity (V_0_) as a function of TMB concentration; (B) Lineweaver–Burk plot of the data in (A); (C) Michaelis–Menten plot of V_0_ as a function of H_2_O_2_ concentration; and (D) Lineweaver–Burk plot of the data in (C).

In the absence of NaCl, CuNW exhibited higher peroxidase-like activity toward TMB than CuNS, with a 2.1-fold greater *V*_max_ (157.60 nM/s vs 74.16 nM/s), indicating a more efficient catalytic process. In contrast, CuNS showed a 4.1-fold lower K_m_ (0.37 mM vs 1.52 mM), suggesting a higher affinity of CuNS for the TMB substrate. The addition of NaCl had distinct effects on the two morphologies. Specifically, NaCl enhanced *V*_max_ 2.3-fold for CuNS and 2.0-fold for CuNW, indicating an increase in the maximum speed of the catalytic reaction. However, the effects on K_m_ were more nuanced, with NaCl increasing it 1.5-fold for CuNS while decreasing it 1.3-fold for CuNW. As for H_2_O_2_, CuNS exhibited higher activity than CuNW, with a 1.2-fold greater *V*_max_ (181.50 nM/s vs 150.80 nM/s), suggesting a higher maximum speed for H_2_O_2_ decomposition. However, CuNW showed a 1.1-fold higher K_m_ (107.43 mM vs 120.20 mM), suggesting a lower affinity for H_2_O_2_. In the presence of chloride ions, the *V*_max_ values for both nanostructures increased, with CuNS exhibiting a 1.4-fold enhancement and CuNW showing a 1.3-fold increase. Meanwhile, the K_m_ values decreased, with CuNS displaying a 1.5-fold reduction and CuNW demonstrating a 2.2-fold decrease.

The modulation of kinetic parameters by chloride ions reveals significant variations in both K_m_ and *V*_max_, leading to changes in the turnover frequency *k*_cat_ (*k*_cat_ = *V*_max_/[E]_t_, where [E]_t_ is the total amount of enzyme active sites) and catalytic efficiency (*k*_cat_/K_m_). A comprehensive comparison of the apparent kinetic parameters (*V*_max_, K_m_, *k*_cat_, and *k*_cat_/K_m_) is presented in Figure 3 and summarized in Table 1. To facilitate a direct comparison between the nanostructures, we consider each Cu atom as a potential active site, thereby avoiding disparities between particle and enzyme numbers.^25^ In all assays, a consistent Cu concentration of 10 μg/mL was used, corresponding to a Cu atom concentration of 1.58 × 10^–4^ M, which was employed as [E]_t_ in the calculation of *k*_cat_. In the absence of NaCl, the *k*_cat_ value of CuNW is 2.1-fold higher than that of CuNS for TMB, whereas the *k*_cat_ values for H_2_O_2_ are comparable for both nanostructures. The addition of NaCl results in increased *k*_cat_ values for both substrates, with a more pronounced effect on TMB. This suggests that an additional mechanism involving chloride ions accelerates the oxidation of TMB.

**Figure 3:**
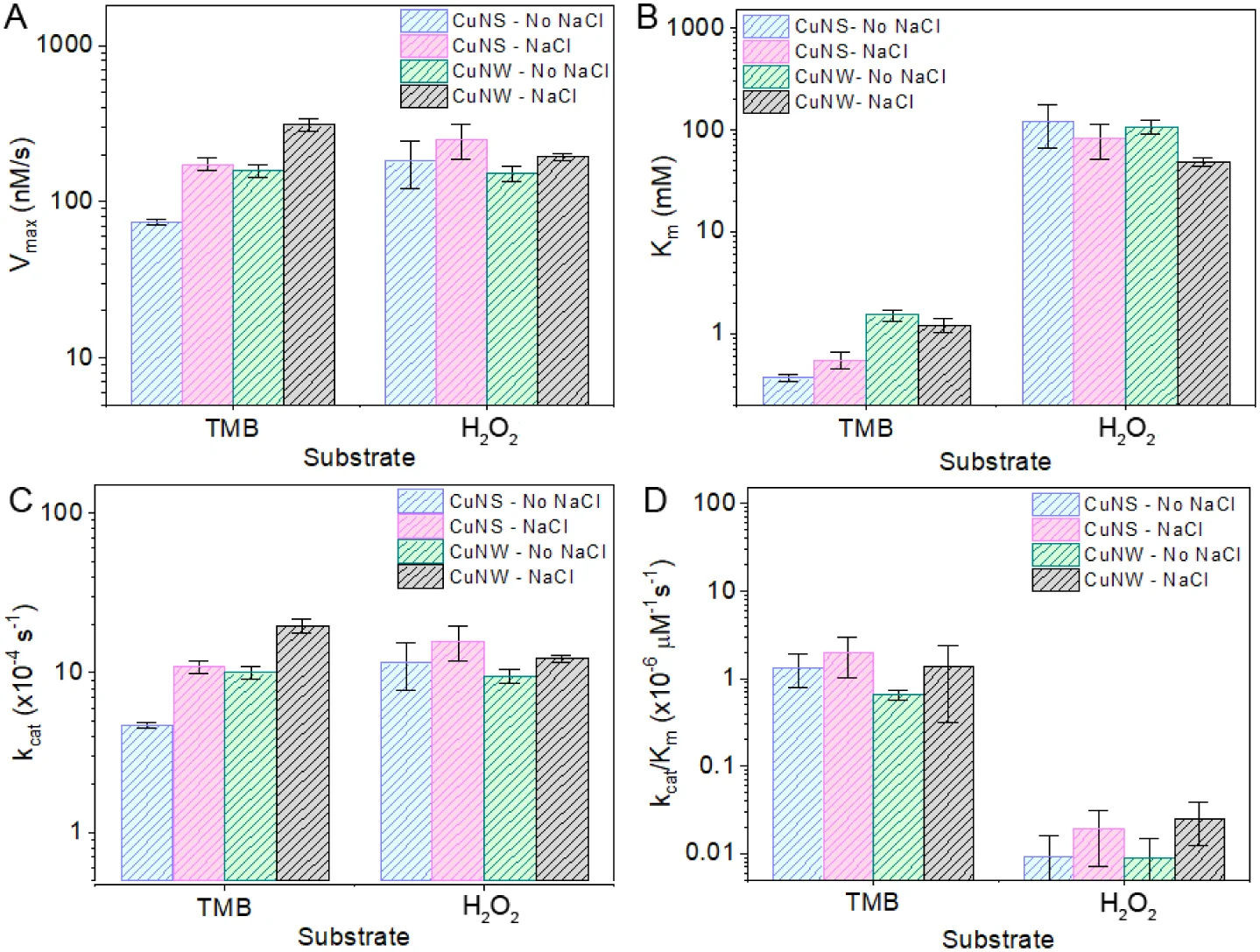
Comparison of the apparent kinetic parameters derived from the Michaelis–Menten mechanism for TMB oxidation on CuNS and CuNW with and without NaCl: (A) *V*_max_; (B) K_m_; (C) *k*_cat_; and (D) *k*_cat_/K_m_. Error bars represent the standard errors of the fit from the average of three independent experiments.

The high affinity of TMB for the Cu nanostructures, as indicated by the low K_m_ values, results in a significantly higher catalytic efficiency *k*_cat_/K_m_ for TMB compared with that for H_2_O_2_. This observation is consistent with a peroxidase-like or Fenton-like reaction pathway, where Cu species catalyze the decomposition of H_2_O_2_ to generate hydroxyl radicals, which subsequently oxidize TMB to form oxidized TMB (oxi-TMB). The resulting blue color of oxi-TMB enabled sensitive colorimetric detection.

The proposed reaction mechanism involved in the above process is illustrated in Figure 4. The Cu nanostructures, comprising a pure Cu core and a Cu_2_O shell, enable a catalytic cycle associated with Cu­(I) and Cu­(II) species, while Cu(0) serves as a sacrificial reservoir. This suggests that the same mechanistic principles that govern the interaction between H_2_O_2_ and Cu_2_O thin films on single-crystal Cu can be applied to the nanostructures, implying a similar underlying catalytic mechanism.^26^ The Cu­(I)/Cu­(II) catalytic cycle can be described by the following steps, represented by balanced eqs 1–3.

1 $$\mathrm{Cu}\left(I\right)\;(s)+H_{2}O_{2}\left(\mathrm{aq}\right)\to \mathrm{Cu}\left(\mathrm{II}\right)\left(s\right)+{\mathrm{OH}}^{-}*+•\mathrm{OH}*$$

2 $$•\mathrm{OH}*+H_{2}O_{2}\left(\mathrm{aq}\right)\to H_{2}O\left(l\right)+•\mathrm{OOH}*$$

3 $$\mathrm{Cu}\left(\mathrm{II}\right)\left(s\right)+•\mathrm{OOH}*\to \mathrm{Cu}\left(I\right)\left(s\right)+O_{2}\left(g\right)+H^{+}*$$

**Figure 4:**
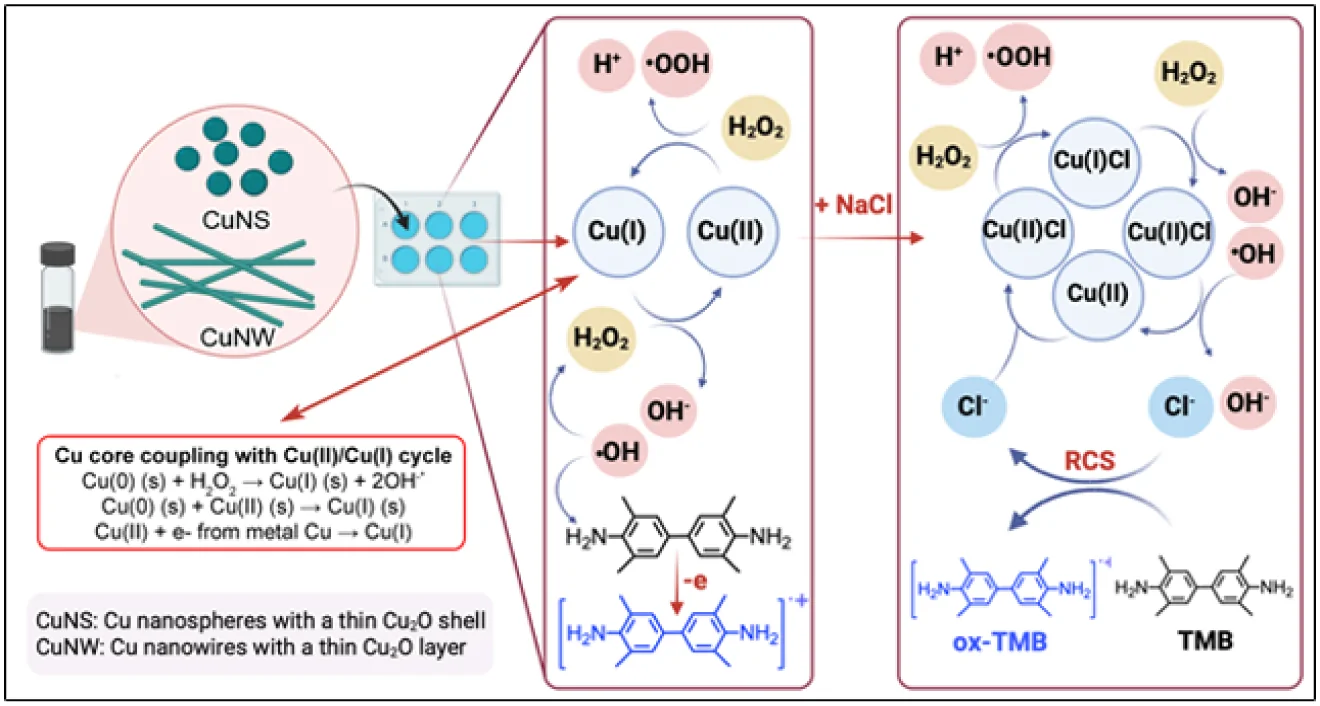
Schematic illustration of the TMB oxidation on CuNS and CuNW nanozymes.

The surface adsorbed OH^–*^ and H^+*^ could combine to form H_2_O (OH^–*^ + H^+*^ → H_2_O), giving the net reaction: 2H_2_O_2_ → H_2_O + O_2_. The Cu­(I)/Cu­(II) catalytic material is regenerative, allowing for repeated use. On the other hand, the Cu(0) in the nanostructures can couple with the Cu­(I)/Cu­(II) catalytic cycle by the following reactions:

4 $$\mathrm{metal}\mathrm{corrosion:}\;\mathrm{Cu}\left(0\right)\left(s\right)+H_{2}O_{2}\to \mathrm{Cu}\left(I\right)\left(s\right)+{\mathrm{2OH}}^{-}*$$

5 disproportionation:Cu(0)(s)+Cu(II)(s)→Cu(I)(s)

6 $$\mathrm{galvanic}/\mathrm{electronic}\;\mathrm{coupling:}\;\mathrm{Cu}\left(\mathrm{II}\right)+e^{-}\;\mathrm{from}\;\mathrm{metal}\;\mathrm{Cu}\to \mathrm{Cu}\left(I\right)$$

These reactions persist as long as bare metal remains exposed, and the oxide layers do not exceed a thickness of approximately 100 nm, at which point the metal becomes buried. Although ICP-MS results showed that 83% and 67% of the total Cu were present in the supernatant after the reactions for CuNS and CuNW, respectively, suggesting a potential contribution from homogeneous catalysis due to dissolved Cu, this does not preclude the occurrence of heterogeneous catalysis. Our TEM and XPS analyses provide evidence for the material evolution proposed in these reaction mechanisms. The TEM image in Figure 5A shows that CuNSs undergo oxidation and become hollow due to the Kirkendall effect.^27^ The Cu 2p XPS and Cu LMM spectra show that the surface of CuNS is composed of Cu and Cu_2_O before reaction, whereas after reaction, it is primarily covered by CuO and Cu_2_O (Figure 5B,C). In contrast, CuNW retains its one-dimensional structure, featuring partly hollow interiors and a reconstructed rough surface, as shown in Figure 5D. The surface changes of CuNW are similar to those of CuNS, with a dominance of Cu/Cu_2_O before reaction and a shift to CuO/Cu_2_O after reaction, as indicated by the XPS spectra in Figure 5E and F. This disparity in conversion has profound implications for their catalytic efficiency, as evidenced by the similar catalytic performance of CuNS and Cu_2_O/Cu nanoparticles (Figure S3, with results listed in Table 1), in contrast to the lower catalytic efficiency exhibited by CuNW.

**Figure 5:**
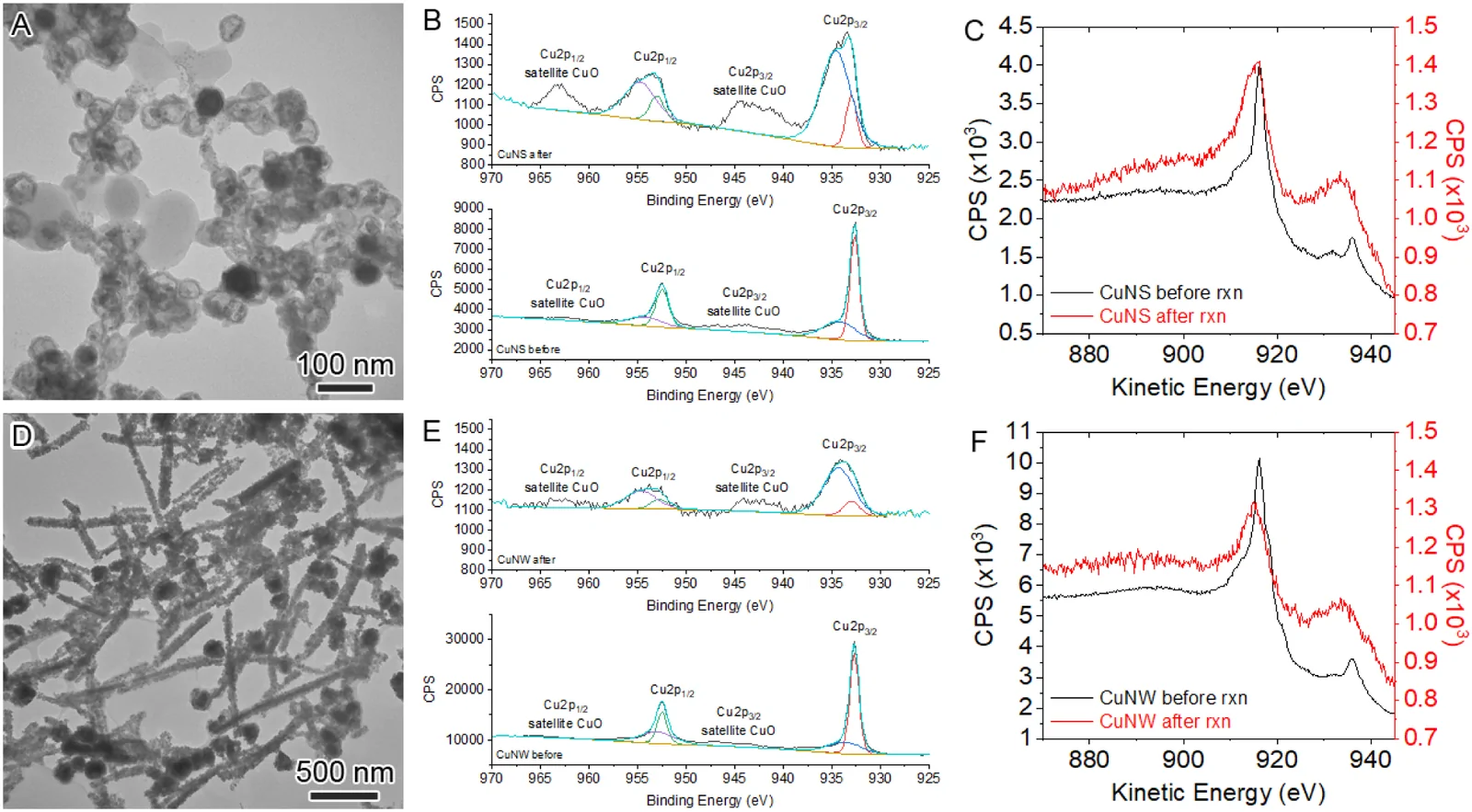
(A–C) Characterization of CuNS: (A) TEM image after the reaction; (B) Cu 2p XPS spectra after the reaction; and (C) Cu LMM spectra before and after reaction. (D–F) Characterization of CuNW: (D) TEM image after the reaction; (E) Cu 2p XPS spectra after the reaction; and (F) Cu LMM spectra before and after reaction.

We further carried out kinetic experiments in the temperature range of 22 to 42 °C at a TMB concentration of 20 μM. Since the substrate concentration is much larger than its K_m_, V_0_ can be used to approximate *V*_max_ which is proportional to *k*_cat_. Using Arrhenius plots, the activation energy (*E*_a_) was calculated to be *E*_a_(CuNW) (14.30 kJ/mol) < *E*_a_(CuNS) (15.54 kJ/mol) (Figure 6A). The presence of Cu(0) in the nanostructures can give rise to freshly generated Cu_2_O, which in turn can lower the activation energy for TMB oxidation. This suggests that the dynamic interplay between Cu(0) and Cu_2_O plays a crucial role in modulating the catalytic activity of the nanostructures, with significant consequences for their overall performance. Terephthalic acid was used as a probe to detect the formation of •OH radicals. Compared to the control, the fluorescence intensity of both CuNS and CuNW increased significantly after 15 min of reaction (Figure 6B). In addition, isopropanol was employed as a radical scavenger in the reaction mixture. As the concentration of isopropanol increased, the absorbance intensity of oxidized TMB decreased dramatically (Figure 6C). These results support the involvement of •OH radicals in the proposed mechanism outlined in Figure 4.

**Figure 6:**
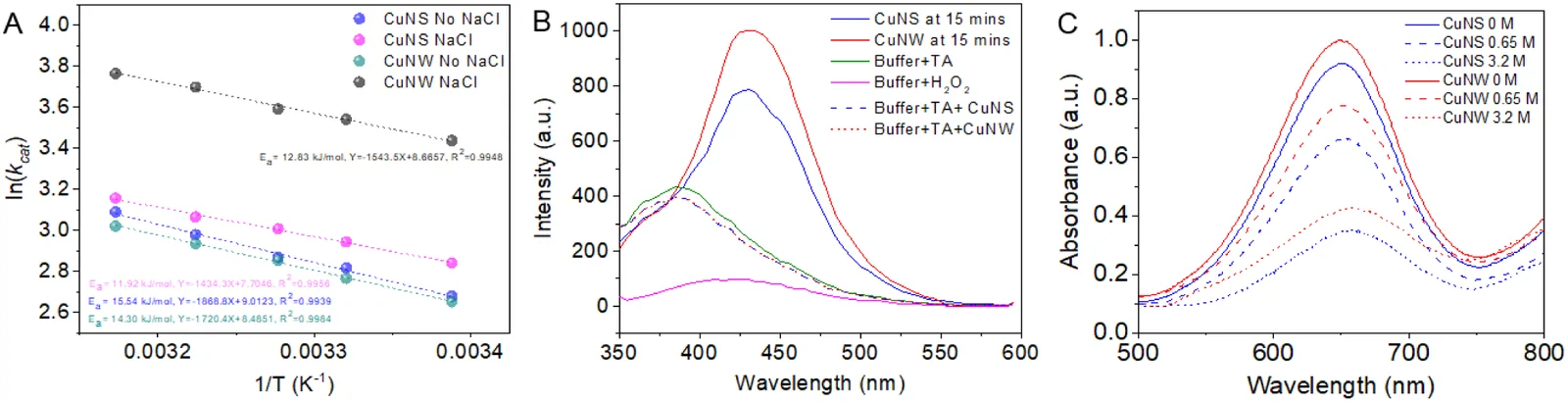
(A) Arrhenius plots of ln­(*k*_cat_) versus 1/T to derive the activation energy (*E*_a_) for TMB oxidation on CuNS and CuNW nanozymes with and without NaCl. (B) Fluorescence emission spectra of terephthalic acid in the CuNS- and CuNW-catalyzed TMB oxidation reactions, compared to controls. (C) UV–vis absorption spectra of the CuNS- and CuNW-catalyzed TMB oxidation reactions after addition of isopropanol, including controls.

The observed chloride-induced modulation of H_2_O_2_ kinetics can be attributed to a direct chemical role for chloride in the Cu-catalyzed Fenton cycle,^11,12,28^ rather than a passive ionic strength effect. Chloride ions accelerate Cu-based Fenton chemistry by coordinating with Cu­(II) to form a [CuCl]^+^ complex, which lowers the activation energy for H_2_O_2_ decomposition and stabilizes Cu­(I) relative to Cu­(II) through preferential Cl^–^ binding to the reduced state. This shift in the Cu­(II)/Cu­(I) redox potential accelerates the rate-limiting Cu­(II) → Cu­(I) → Cu­(II) cycling step, increasing the overall rate of ROS generation. Furthermore, chloride is oxidized during the reaction to form reactive chlorine species (RCS) through a series of steps:

7 $$•\mathrm{OH}*+{\mathrm{Cl}}^{-}\left(\mathrm{aq}\right)\to H_{2}O\left(l\right)+•{\mathrm{ClOH}}^{-}*$$

8 $${•\mathrm{ClOH}}^{-}*+H^{+}\left(\mathrm{aq}\right)\to \mathrm{Cl•}*+H_{2}O\left(l\right)$$

9 $$\mathrm{Cl•}*+{\mathrm{Cl}}^{-}\left(\mathrm{aq}\right)\to {\mathrm{Cl}}_{2}{•}^{-}*$$

These RCS can directly oxidize TMB, independent of hydroxyl radicals, providing a secondary route to signal amplification, as illustrated in Figure 4. Although Cl_2_•^–^ (E° ≈ 2.0 V) is a weaker oxidant than •OH (E° ≈ 2.8 V),^29^ it reacts more selectively and at substantially lower rate constants.^30^ This affords it a longer lifetime, allowing it to accumulate to higher steady-state concentrations^31^ that facilitate TMB oxidation. The addition of NaCl enhances the catalytic activity of both Cu nanostructures, with comparable catalytic efficiencies (*k*_cat_/K_m_ values of 1.99 for CuNS and 1.62 for CuNW). Replacing NaCl with NaNO_3_ and Na_2_SO_4_ do not improve the catalytic activity of the Cu nanostructures (Figure S4), suggesting that chloride-specific coordination chemistry enhances the catalytic activities. The *E*_a_ values for the reactions in the presence of NaCl are significantly lower than those without NaCl (Figure 6A). The *E*_a_ value for CuNS decreases from 15.54 to 11.92 kJ/mol upon addition of NaCl, while the activation energy of CuNW drops from 14.30 to 12.83 kJ/mol, demonstrating the significant role of chloride ions in modulating catalytic activity.

To investigate metallic Cu as a nanozyme, we stabilized Cu with Au and examined their peroxidase-like activity. The Cu_3_Au alloy and intermetallic nanoparticles were synthesized using our previously established method.^24^ TEM images (Figure 7A and B) show that both nanoparticles are rod-shaped, with average dimensions of 7.6 nm × 15.6 nm and 6.8 nm × 12.6 nm for the alloy and the intermetallic, respectively. The XRD pattern of the Cu_3_Au alloy (Figure 7C) corresponds to a disordered fcc structure, with characteristic diffraction peaks at 2θ of 41.32°, 47.99°, 70.77°, and 85.55°, which can be indexed to the (111), (200), (220), and (311) crystal planes, respectively. The expected peak positions were calculated using Vegard’s law based on the lattice parameters of pure Au and Cu. In contrast, the diffraction peaks of the Cu_3_Au intermetallic (Figure 7D) exhibit characteristic reflections at 2θ of 23.83°, 34.05°, 41.91°, 48.68°, 54.86°, 60.66°, 71.44°, 76.38°, 81.83°, and 86.28°, which can be indexed to the (100), (110), (111), (200), (210), (211), (220), (300), (310), and (311) planes, respectively.

**Figure 7:**
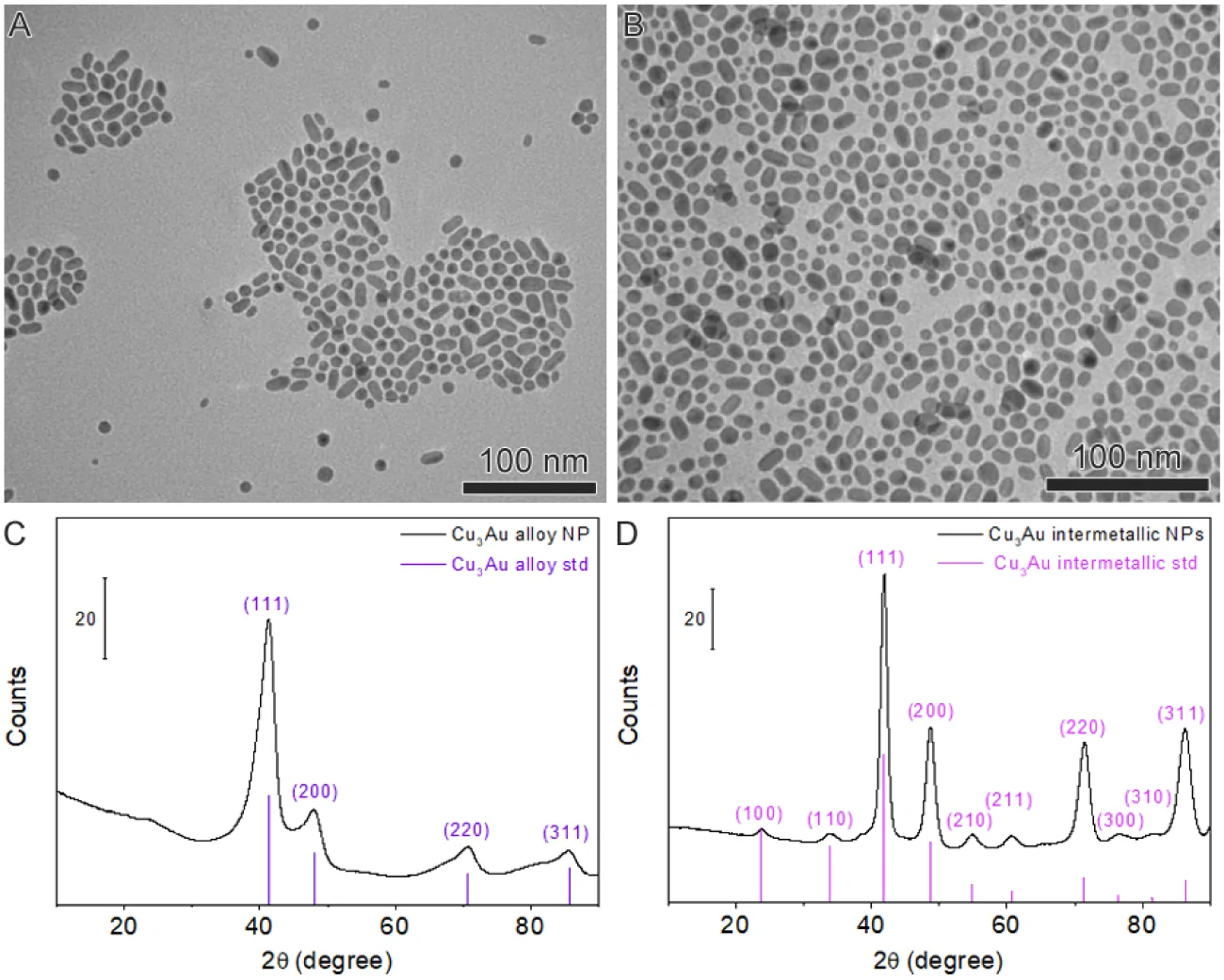
(A) TEM image of Cu_3_Au alloy nanoparticles, with an average diameter of 7.6 ± 1.0 nm and an average length of 15.6 ± 3.6 nm, (B) TEM image of Cu_3_Au intermetallic nanoparticles, with an average diameter of 6.8 ± 1.5 nm and an average length of 12.6 ± 3.0 nm, (C, D) XRD pattern of PEGylated Cu_3_Au alloy and intermetallic nanoparticles after the ligand exchange process. The purple indexes correspond to the Cu_3_Au alloy structure, derived from Vegard’s law based on Cu (MP-30) and Au (MP-81) standards. The magenta indexes correspond to the Cu_3_Au intermetallic standard (MP-2258).

The catalytic kinetics of the Cu_3_Au alloy and intermetallic nanoparticles were evaluated using Michaelis–Menten and Lineweaver–Burk analyses. By varying TMB concentrations at a fixed H_2_O_2_ concentration or vice versa (Figure 8), characteristic Michaelis–Menten behavior was observed, with corresponding Lineweaver–Burk plots showing good linearity. This observation indicates that the catalytic reactions follow the classical steady-state enzyme kinetics. The apparent kinetic data using hyperbolic fits to the Michaelis–Menten plots are summarized in Table 2.

**Table 2:** Summary of Apparent Kinetic Parameters (K_m_, *V*_max_, *k*_cat_) for TMB Oxidation Catalyzed by Cu_3_Au Alloy and Cu_3_Au Intermetallic Nanozymes with and without NaCl^a^

		K_m_ (mM)		*V*_max_ (nM/s)		*k*_cat_ (×10^–4^ s^–1^)		*k*_cat_/K_m_ (×10^–6^ μM^–1^ s^–1^)	
Nanozyme	Substrate	No NaCl	NaCl	No NaCl	NaCl	No NaCl	NaCl	No NaCl	NaCl
Cu_3_Au alloy	TMB	0.61	2.43	38.00	191.66	3.68	18.56	0.60	0.76
	H_2_O_2_	69.30	117.74	77.83	136.00	7.54	13.20	0.01	0.01
Cu_3_Au intermetallic	TMB	1.58	2.19	21.00	64.00	2.03	6.20	0.13	0.28
	H_2_O_2_	146.20	79.43	24.50	21.60	2.37	2.10	0.002	0.002

a *k*_cat_ was calculated based on the sum of the Cu and Au atom concentrations (1.03 × 10^–4^ M).

**Figure 8:**
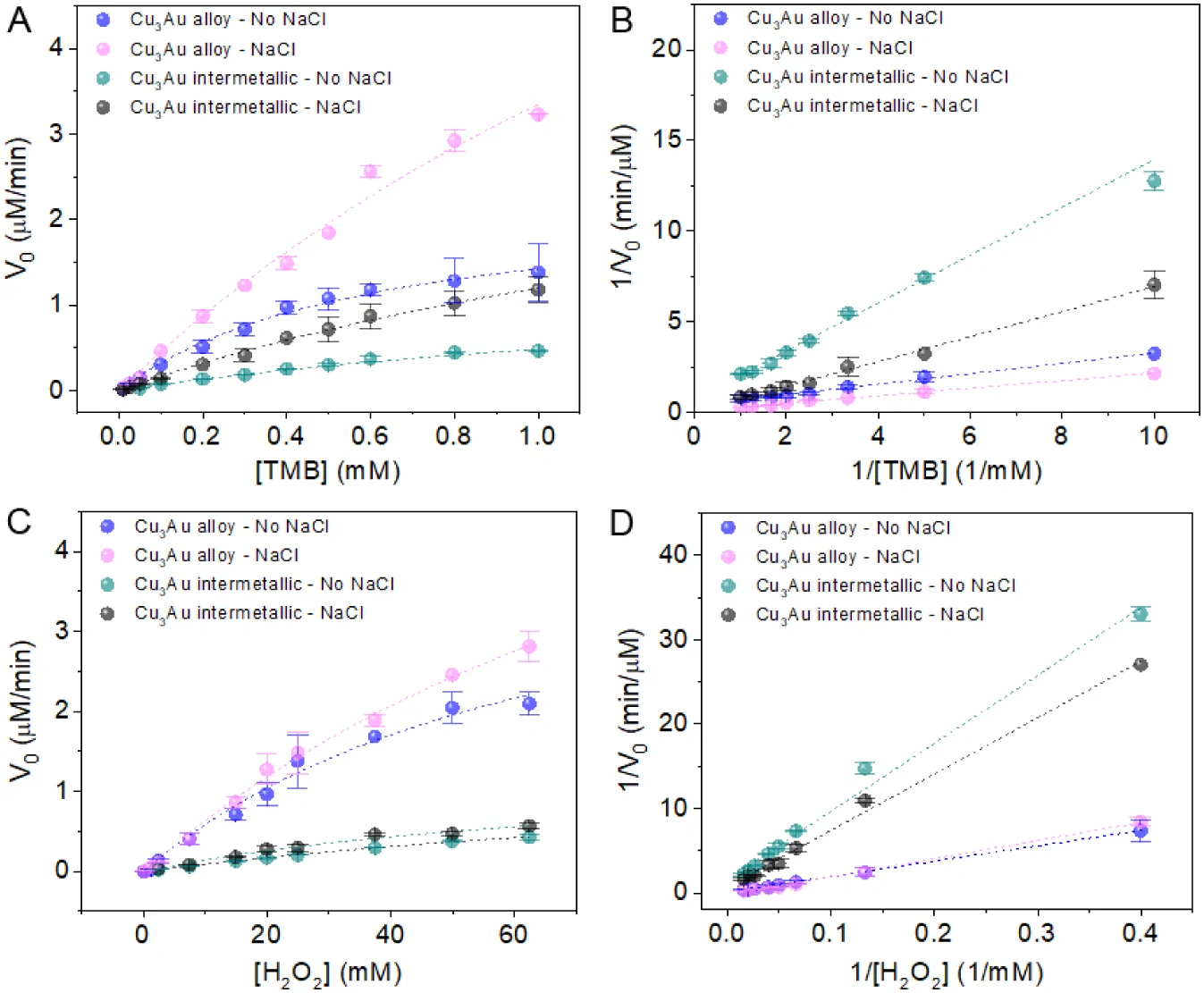
Steady-state kinetic analysis for TMB oxidation catalyzed by Cu_3_Au alloy and intermetallic nanozymes in the presence and absence of NaCl: (A) Michaelis–Menten plot of initial reaction velocity (V_0_) as a function of TMB concentration; (B) Lineweaver–Burk plot of the data in (A); (C) Michaelis–Menten plot of V_0_ as a function of H_2_O_2_ concentration; and (D) Lineweaver–Burk plot of the data in (C).

In the absence of NaCl, the K_m_ values for the Cu_3_Au alloy were 0.61 mM and 69.30 mM toward TMB and H_2_O_2_, respectively, which are comparable to those of CuNS. The comparable substrate affinity can be attributed to the Cu-rich composition of Cu_3_Au, which results in comparable binding properties for both of the substrates. However, the *V*_max_ values for the Cu_3_Au alloy were 38 nM/s and 77.83 nM/s toward TMB and H_2_O_2_, respectively, representing decreases of 49% and 57% compared to CuNS. In contrast, the Cu_3_Au intermetallic exhibited weaker substrate binding with 2.6-fold higher k_m_ for TMB and 2.1-fold higher K_m_ for H_2_O_2_. As inferred from its binding affinity, the intermetallic appears to have a lower surface concentration of Cu compared with the alloy. This is supported by the ICP-MS results, which show that 26% and 16% of the total Cu were present in the supernatant after the reactions for the alloy and intermetallic, respectively. The *V*_max_ values for the intermetallic were reduced by 50% for TMB and by 70% for H_2_O_2_ compared with those of the alloy. Upon addition of NaCl, the affinities of both Cu_3_Au nanoenzymes for TMB decreased, whereas their *V*_max_ values increased. For the alloy, the affinity for H_2_O_2_ and TMB decreased 1.7-fold and 4.0-fold, respectively, whereas the intermetallic exhibited an increase in affinity of 1.8-fold for H_2_O_2_ and a decrease in affinity of 1.4-fold for TMB. The *V*_max_ for the alloy increased significantly, reaching 191.6 nM/s, but for the intermetallic it increased to 64 nM/s.

A comprehensive comparison of the apparent kinetic parameters (*V*_max_, K_m_, *k*_cat_, and *k*_cat_/K_m_) for Cu_3_Au alloy and intermetallic nanoparticles is presented in Figure 9 and summarized in Table 2. In calculating these values, we assume that each Cu or Au atom serves as a potential active site, corresponding to a total metal concentration of 1.03 × 10^–4^ M in all assays. In the absence of NaCl, the *k*_cat_ value for the alloy is 1.8-fold higher than that of the intermetallic for TMB, whereas the *k*_cat_ value for H_2_O_2_ is 3.2-fold higher for the alloy compared to the intermetallic. The addition of NaCl results in increased *k*_cat_ values for the alloy, with a more pronounced effect on TMB (5-fold increase) than H_2_O_2_ (1.8-fold increase), suggesting that chloride ions accelerate the oxidation of TMB.

**Figure 9:**
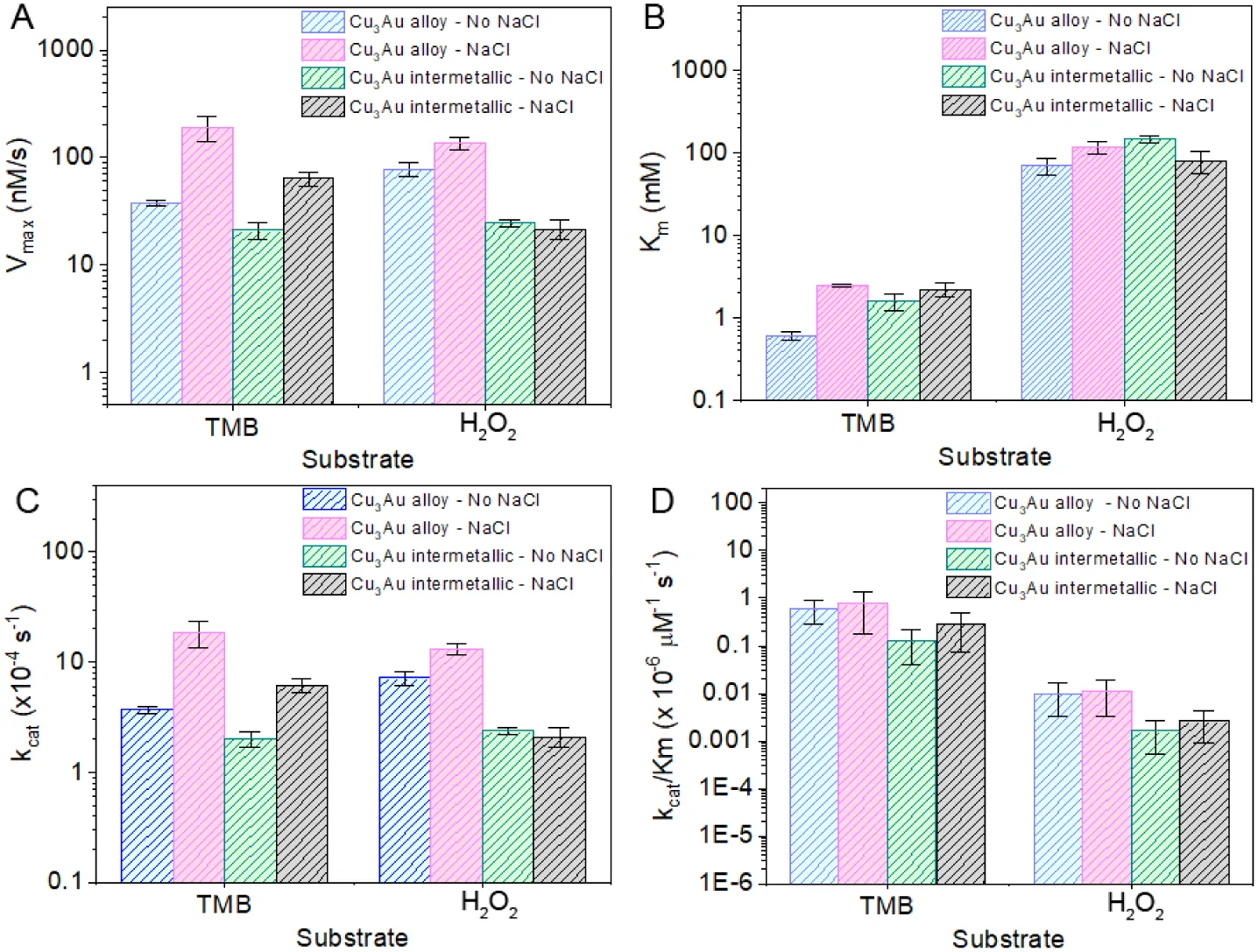
Comparison of the apparent kinetic parameters derived from the Michaelis–Menten mechanism for TMB oxidation on Cu_3_Au alloy and intermetallic nanoparticles with and without NaCl: (A) *V*_max_; (B) K_m_; (C) *k*_cat_; and (D) *k*_cat_/K_m_. Error bars represent the standard errors of the fit from the average of three independent experiments.

To compare the catalytic efficiency of the alloy and intermetallic for TMB and H_2_O_2_ in the absence and presence of NaCl, we normalized the *k*_cat_ values to K_m_. Under NaCl-free conditions, the catalytic efficiency of the alloy is 4.6-fold and 5-fold higher than that of the intermetallic for TMB and H_2_O_2_, respectively. Upon addition of NaCl, the catalytic efficiency of the alloy increases by 27% for TMB but remains the same for H_2_O_2_. In contrast, NaCl addition enhances the catalytic efficiency of the intermetallic by 100% for TMB while having no effect on its efficiency for H_2_O_2_.

The reaction pathways for Cu_3_Au alloy and intermetallic nanoparticles could involve a combination of hydroxyl radical-based Fenton^17^ and direct electron transfer mechanisms.^20–22^ Cu has a stronger tendency to bind oxygen than Au, which is a crucial factor in determining the catalytic activity of these materials. The distribution of Cu to Au substantially impacts the affinity for H_2_O_2_, thereby affecting the catalytic efficiency. Based on the Fenton reaction mechanism, alloying Cu with Au stabilizes Au and diminishes the Cu(0)-coupling mechanism that facilitates the reaction, leading to a decrease in *k*_cat_ for this reaction pathway. However, the *k*_cat_ values of CuNS and Cu_3_Au for TMB are surprisingly similar, suggesting that Cu_3_Au could undergo a direct electron transfer mechanism through the H-assisted O–O cleavage H_2_O_2_^*^ + H^*^ → H_2_O^*^ + OH^*^ proposed for noble metal such as Au.^21^ It is worth noting that Cu_3_Au nanoparticles are markedly more active than Au nanoparticles (Figure S5), consistent with previous reports.^19^ This enhancement can be attributed to Cu, which lowers the OH^*^ generation barrier on the bimetallic surface in the direct electron transfer mechanism.

The stronger H_2_O_2_ binding (lower K_m_) of the alloy arises from its less stable Cu compared to the intermetallic^32,33^ and thus more chemically accessible.^34^ This interpretation is consistent with our previous study^35^ and others^36^ on the dealloying of Cu_3_Au, in which the more negative critical potential and faster dealloying of the alloy versus the ordered intermetallic indicate that Cu is less stable. The atomic distribution of Cu and Au in the crystal structure of the nanoparticles also significantly impacts the *k*_cat_ values for both H_2_O_2_ and TMB, resulting in 3.2-fold and 1.8-fold higher turnover rates on Cu_3_Au alloy compared to Cu_3_Au intermetallic. This trend suggests that the random arrangement of Cu and Au atoms in the alloy enhances the catalytic activity, whereas the intermetallic ordering structure leads to a less-efficient catalyst.

Our TEM, XPS, and ICP-MS analyses, combined with the terephthalic acid and isopropanol experiments, provide evidence for hydroxyl radical-based Fenton and direct electron transfer mechanisms. In contrast to Cu nanostructures, the TEM images in Figure 10A and D show that both the alloy and intermetallic nanoparticles remain solid particles after the reaction, suggesting that negligible oxidation occurs. This observation is consistent with the Cu 2p XPS (Figure 10B and E) and Cu LMM spectra (Figure 11C and F), which indicate that the surface of both the alloy and intermetallic nanoparticles is primarily composed of metallic Cu after the reaction. It is worth noting that the Cu 2p XPS spectra exhibit CuO components in both the alloy and intermetallic nanoparticles before the reaction, suggesting that these nanoparticles might be covered by a very thin layer of oxidized Cu. ICP-MS results reveal that 26% and 16% of the total Cu are present in the supernatant after the reaction for the alloy and intermetallic nanoparticles, respectively. These findings suggest an inhomogeneous distribution of Cu and Au on the surfaces of both the alloy and intermetallic nanoparticles with more pronounced inhomogeneity observed for the alloy.

**Figure 10:**
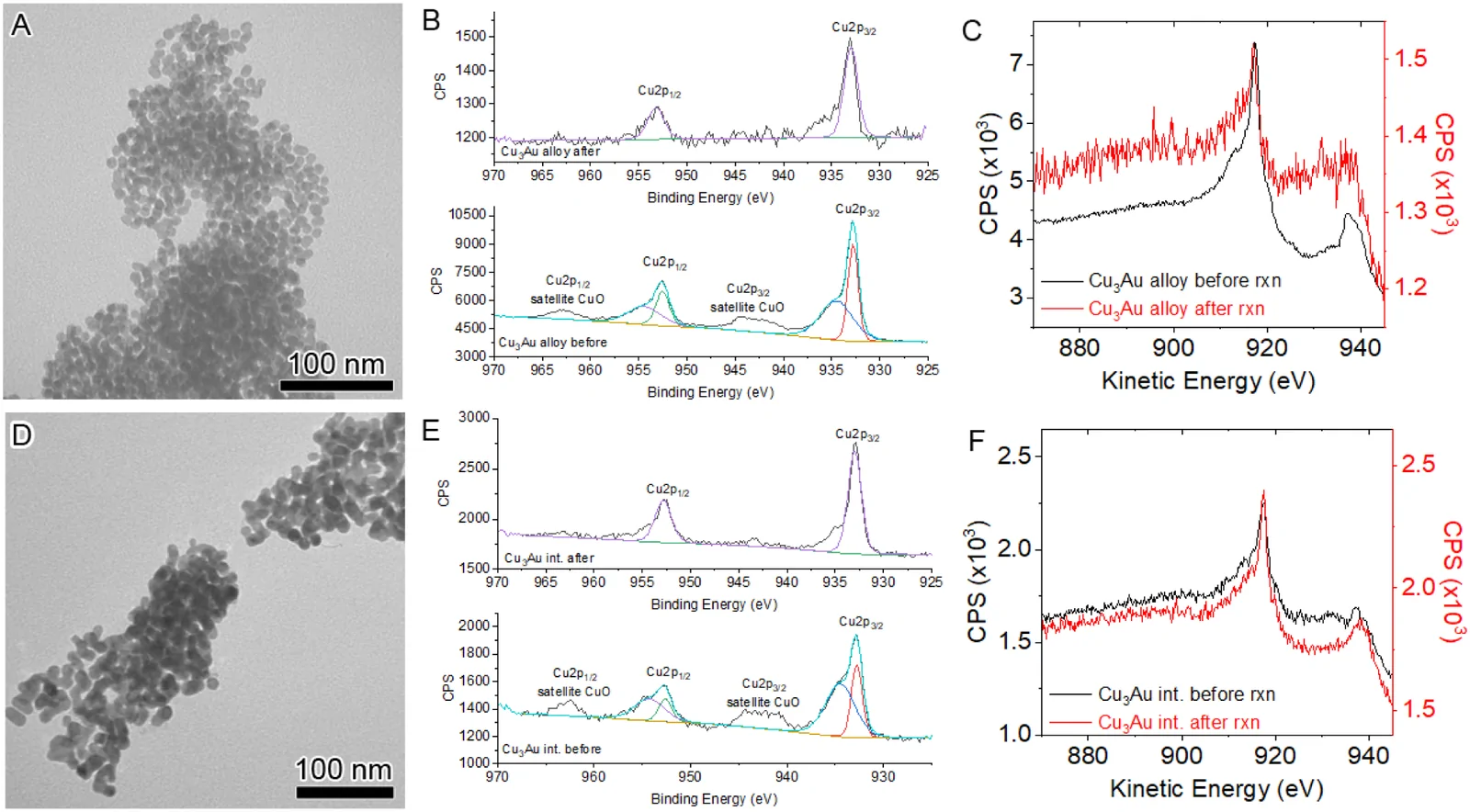
(A–C) Characterization of Cu_3_Au alloy: (A) TEM image after the reaction; (B) Cu 2p XPS spectra after the reaction; and (C) Cu LMM spectra before and after reaction. (D–F) Characterization of Cu_3_Au intermetallic: (A) TEM image after the reaction; (B) Cu 2p XPS spectra after the reaction; and (C) Cu LMM spectra before and after reaction.

**Figure 11:**
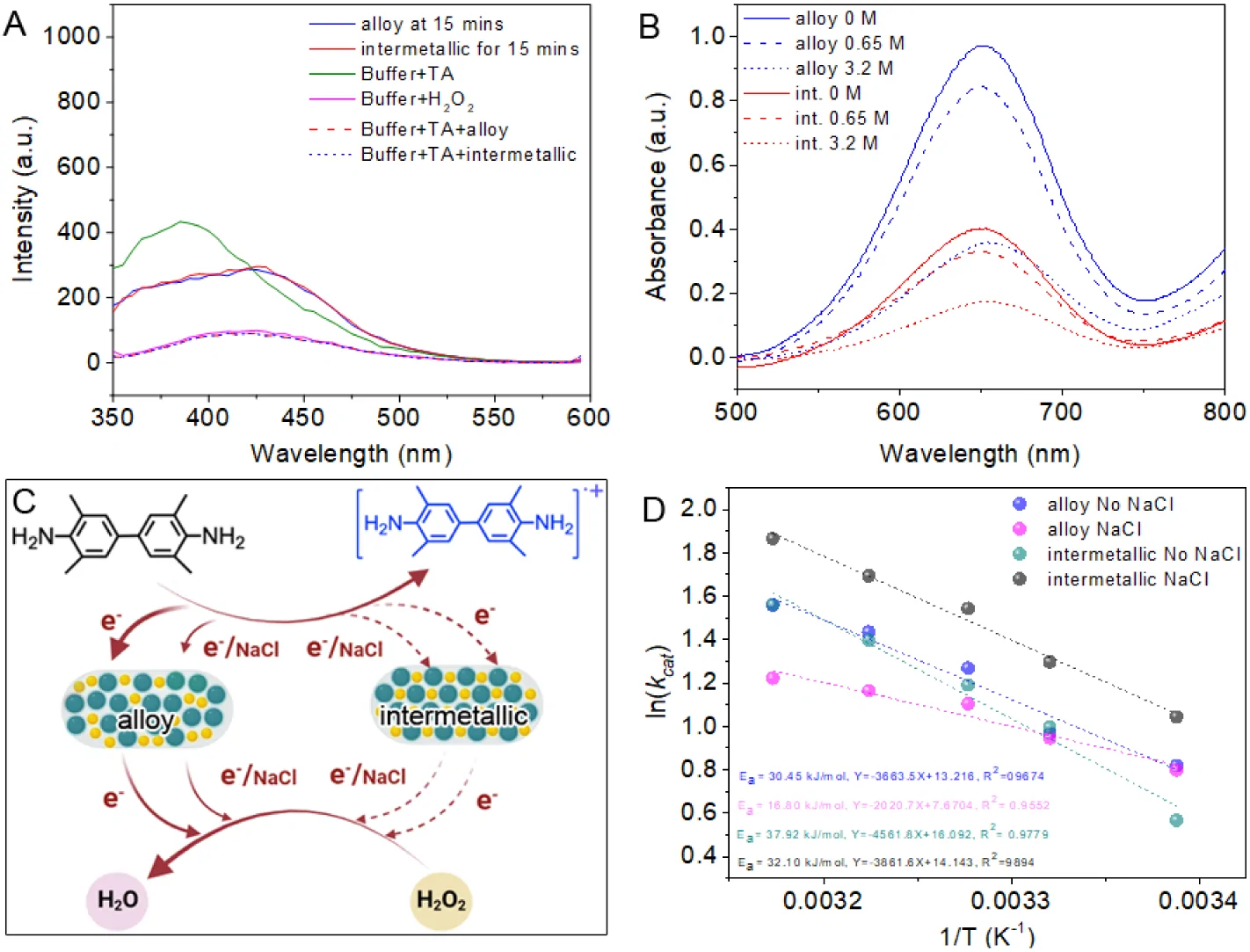
(A) Fluorescence emission spectra of terephthalic acid in the alloy- and intermetallic-catalyzed TMB oxidation reactions, compared to controls. (B) UV–vis absorption spectra of the alloy- and intermetallic-catalyzed TMB oxidation reactions after the addition of isopropanol, including controls. (C) Schematic illustration of TMB oxidation on CuNS and CuNW nanozymes. (D) Arrhenius plots of ln­(*k*_cat_) versus 1/T to derive the activation energy (*E*_a_) for TMB oxidation on Cu_3_Au alloy and intermetallic nanozymes with and without NaCl.

In the terephthalic acid experiments, compared to the control, the fluorescence intensities of both the alloy and intermetallic nanoparticles remain similar after 15 min of reaction (Figure 11A). However, when isopropanol was used as a radical scavenger in the reaction mixture, the absorbance intensity of oxidized TMB decreased as the concentration of isopropanol increased (Figure 11B), but the decrease was not as pronounced as that of the Cu nanostructures. These results suggest that the alloy and intermetallic nanoparticles involve both a direct electron transfer mechanism and a hydroxyl radical mechanism due to the presence of both the metallic Cu/Au and oxidized Cu. Since both mechanisms involve electron transfer, we simplified the proposed mechanism as illustrated in Figure 11C.

We also carried out a temperature-dependent kinetic study to calculate the *E*_a_ of the reactions, as shown in Figure 11D. The *E*_a_ values for the alloy and intermetallic are 30.45 and 37.92 kJ/mol, respectively, indicating that the intermetallic requires more energy to initiate the catalytic reaction. This difference in the *E*_a_ values suggests that the alloy is more kinetically favorable for the reaction. Upon addition of chloride ions, the local coordination environment of both Cu and Au is altered, leading to decreased *E*_a_ values for both forms (16.80 and 32.10 kJ/mol). This reduction in *E*_a_ indicates that the presence of chloride ions facilitates the catalytic reaction by lowering the energy barrier, making it more kinetically favorable. Replacing chloride with nitrate or sulfate has little effects on the catalytic activities of the alloy and intermetallic (Figure S4), also suggesting that chloride has specific effects on the alloy and intermetallic.

## Conclusion

We demonstrated the peroxidase-like activity of Cu-based nanostructures, including CuNS and CuNW, as well as Cu–Au alloys and intermetallic compounds. Our results showed that CuNW exhibited 2.1-fold higher activity toward TMB than CuNS, with a *k*_cat_ of 10 × 10^–4^ s^–1^ in the absence of NaCl. The addition of NaCl enhanced the catalytic activity of both CuNS and CuNW, resulting in increased *k*_cat_ values and decreased *E*_a_. The *E*_a_ value for CuNS decreased from 15.54 to 11.92 kJ/mol upon addition of NaCl, while the activation energy of CuNW dropped from 14.30 to 12.83 kJ/mol. The mechanism involves a Cu­(I)/Cu­(II) catalytic cycle, where Cu metal coupling plays a crucial role in facilitating the reaction, and chloride ions accelerate the reaction by coordinating with Cu­(II) to form a [CuCl]^+^ complex. Our findings also revealed that the Cu_3_Au alloy and intermetallic nanoparticles exhibited distinct substrate-binding properties and catalytic activities, with the alloy showing higher *k*_cat_ values for TMB oxidation than the intermetallic (3.68 × 10^–4^ s^–1^ vs 2.03 × 10^–4^ s^–1^). The reaction mechanism involves both direct electron transfer and hydroxyl radicals on the metal surface. The stability of Cu to Au in the alloy and intermetallic substantially impacted the affinity for H_2_O_2_, thereby affecting the catalytic efficiency. The chloride ion also has a positive effect on the catalytic activity. The *E*_a_ values for the Cu_3_Au alloy were 30.45 kJ/mol (without NaCl) and 16.80 kJ/mol (with NaCl), compared to 37.92 kJ/mol (without NaCl) and 32.10 kJ/mol (with NaCl) for the intermetallic. The Cu-based nanostructures can be lyophilized and stored until use. These results provide valuable insights into the development of nonprecious metal-containing catalysts as efficient nanoenzymes for applications including biosensing, biocatalysis, and environmental remediation.

## Experimental Methods

### Chemicals and Materials

Copper 2,4-pentanedionate (Cu­(acac)_2_, 98%), 1-octadencene (ODE, 90%), tri-n-octylphosphine (TOP, 90%), and sodium sulfate (99.0%, ACS) were purchased from Alfa Aesar. Copper­(II) Chloride (CuCl_2_, anhydrous, 98%), 3,3′,5,5′-Tetramethylbenzidine (TMB, 98%), and sulfuric acid (98%, ACS grade) were purchased from Beantown Chemical. Formic acid (99%), and sodium acetate (Na­(ac)_2_, anhydrous ACS grade) were purchased from VWR Chemicals. Hydrogen Peroxide (30%, Electronic grade) was purchased from Avantor Performance Materials. Gold­(III) Chloride hydrate (HAuCl_4_·*x*H_2_O) was purchased from AmBeed. Poly­(ethylene glycol) methyl ether (PEG, *M*_n_ = 2000), oleylamine (OLAM, 70%), and terephthalic acid (TA, 98%) were purchased from Sigma-Aldrich. Sodium nitrate (97%, ACS) was purchased from Thermo Scientific. Chloroform, hexanes, and toluene were purchased from EMD. Ethanol (100%) was purchased from Koptec. Isopropanol was purchased from Macron Chemicals. All chemicals were used as received. Ultrapure water with a resistance of 18 MΩ was used in all experiments.

### Synthesis of Cu Nanospheres (CuNS)

Spherical nanoparticles were synthesized using our previously established method by reducing Cu­(acac)_2_ (52.4 mg, 0.2 mmol) with OLAM in a mixture of ODE (4 mL) and OLAM (1 mL) at 220 °C under argon protection.^37^ The reaction involved purging with argon for 10 min, followed by the addition of TOP (1 mL), and then introducing CO (∼15 mL) vapor generated from formic acid dehydration by sulfuric acid at 200 °C. After 20 min at 220 °C, the product was purified through centrifugation (6000 rpm, 5 min, repeated three times) using an ethanol/toluene mixture (4:1 ratio, 25 mL), and then redispersed in toluene (3 mL).

### Synthesis of Cu Nanowires (CuNW)

Cu nanowires were synthesized based on our previously established method by reducing Cu­(acac)_2_ (0.2 mmol, 52.5 mg) and CuCl_2_ (0.2 mmol, 26.4 mg) in OLAM (5 mL) at 220 °C under argon protection for 50 min.^23^ After the reaction, the mixture was transferred to a 15 mL centrifuge tube, filled with ethanol to 15 mL, and centrifuged at 6000 rpm for 4 min. The product was purified through centrifugation (6000 rpm, 4 min, repeated twice) using an ethanol/toluene mixture (2:13 v/v ratio, 15 mL), and then redispersed in toluene (2 mL).

### Synthesis of Cu_3_Au Nanoparticles

Cu_3_Au nanoparticles were synthesized by our modified procedures.^24^ For the alloy, HAuCl_4_·3H_2_O (19.7 mg, 0.05 mmol) dissolved in OLAM (5 mL) was reacted at 160 °C for 20 min and then cooled to 60 °C before Cu­(acac)_2_ (52.4 mg, 0.2 mmol) dissolved in OLAM (2.5 mL) was injected to the reaction. The reaction mixture was subsequently heated to 180 °C and maintained at this temperature for 40 min. After the reaction, the product was purified through centrifugation (8000 rpm, 4 min, repeated three times) using an ethanol/toluene mixture (2:13 v/v ratio, 15 mL), and then redispersed in toluene (2 mL). For the intermetallic, the same procedure was used except that the reaction mixture was subsequently heated to 280 °C and maintained at this temperature for 40 min.

### Ligand Exchange Process

Particles dispersed in toluene (1 mL) were added to chloroform (10 mL) containing PEG at a concentration of 1 mg/mL. The mixture was stirred at room temperature for 6 h. After completion, the suspension was transferred to a 50 mL centrifuge tube, filled with hexane to 30 mL, and centrifuged at 9000 rpm for 10 min. The product was purified through centrifugation (14,000 rpm, 15 min, repeated three times) using ethanol, and then redispersed in ethanol (3 mL) and stored at 4 °C.

### Characterization

TEM images were taken using a TEM (JEOL JEM-1011) at an acceleration voltage of 100 kV. STEM-HAADF images and EDX elemental maps were obtained using the Thermo Fisher Talos 200X, a 200 keV high-resolution analytical STEM equipped with a four-quadrant energy-dispersive X-ray spectrometer. XRD patterns were recorded using a diffractometer (Rigaku XtaLAB Synergy-S) with a Cu K_α_ radiation source. Elemental concentrations of the samples were measured using inductively coupled plasma mass spectrometry (ICP-MS, Thermo Scientific iCAP Q). X-ray photoelectron spectroscopy (XPS) was performed using a PHI VersaProbe station. The XPS spectra were analyzed using CasaXPS software.

### Kinetic Measurements

Steady-state kinetic assays were performed at room temperature (∼22 °C) in 24-well plates using a Tecan Infinite M200 microplate reader. Sodium acetate buffer (50 mM, pH 4.4) was used to dissolve H_2_O_2_ and TMB at various concentrations. Nanoparticle samples (20 μL, 1 mg/mL) were added to maintain a constant total metal mass of 0.02 mg per reaction. The catalytic oxidation of TMB was monitored by recording absorbance changes at 652 nm over time. Assays with varying H_2_O_2_ and TMB concentrations with 50 μL of each added to 1880 μL of buffer were carried out to determine the apparent kinetic parameters (*V*_max_, K_m_, *k*_cat_, *k*_cat_/K_m_) for these two substrates in the Michaelis–Menten reaction mechanism for different nanozymes. Control experiments were conducted to rule out background signals and spontaneous oxidation.

The absorbance vs time plots were analyzed to determine the initial rate (V_0_) of each reaction, for which only the linear portions of the initial reaction progress curves were considered. The V_0_ values were calculated using the molar absorption coefficient of oxidized TMB at 653 nm (39,000 M^–1^ cm^–1^) and an optical path length of 1 cm. The Michaelis–Menten model was fitted to the plot of V_0_ against substrate concentration ([S]), and the data were also transformed into Lineweaver–Burk double-reciprocal plots (1/V_0_ vs 1/[S]). *E*_a_ values were obtained through Arrhenius plot analysis at a TMB concentration of 20 mM to ensure V_0_ ≈ *V*_max_ at temperatures of 22 °C, 28 °C, 32 °C, 37 °C, and 42 °C.

### Anion Study

A 50 mM sodium acetate buffer was prepared by dissolving 1028 mg of sodium acetate in 250 mL of water. The pH was adjusted to 4.4 by titrating with HCl. For the anion effects, NaCl (287 mg), NaNO_3_ (417 mg), and NaSO_4_ (679 mg) were added to the buffer individually, resulting in three buffer conditions to compare the original buffer with no salt addition. Kinetic measurements were performed in a total reaction volume of 2000 μL. Briefly, 1880 μL of buffer (with the respective salt condition) was combined with 50 μL of 8 mM TMB and 20 μL of Cu-based nanozymes (1 mg/mL). The reaction was initiated by the addition of 50 μL of 0.8 M hydrogen peroxide (H_2_O_2_). The oxidation of TMB was monitored by measuring absorbance spectra using a UV–vis spectrometer, and reaction kinetics were tracked by recording absorbance at 652 nm as a function of time.

### Hydroxyl Radical Study

The hydroxyl radical-generating capacity of the Cu-based nanostructures was assessed using the terephthalic acid (TA) fluorescence probe assay. Briefly, 1910 μL of sodium acetate buffer (50 mM, pH 4.4, no NaCl) was added to a quartz cuvette, followed by 20 μL TA (100 mM), 20 μL of Cu-based nanostructures (1 mg/mL), and 50 μL of H_2_O_2_ (0.8 M) to initiate the reaction (final volume: 2000 μL). The mixture was gently vortexed for 3 s immediately after H_2_O_2_ addition. Fluorescence emission was recorded from 350 to 600 nm using a PerkinElmer LS 55 luminescence spectrometer, with excitation at 300 nm, excitation and emission slit widths of 9 nm, and a scan speed of 300 nm/min. Spectra were collected at 0, 5, 10, and 15 min after H_2_O_2_ addition, with all measurements performed at room temperature. Appropriate controls (TA + buffer only, TA + H_2_O_2_ without nanostructures, and TA + nanostructures without H_2_O_2_) were included to account for background fluorescence and nonspecific TA oxidation.

Radical scavenging experiments were performed using isopropanol (IPA) as a hydroxyl radical scavenger. Assays were conducted in a total reaction volume of 2000 μL, containing 50 μL of 8 mM TMB, 20 μL of Cu-based nanozymes (1 mg/mL), and either 100 or 500 μL of pure isopropanol, with the remaining volume brought up to 2000 μL using 50 mM sodium acetate buffer (pH 4.4, no salt addition). The reaction was initiated by the addition of 50 μL of 0.8 M H_2_O_2_. The final IPA concentration corresponded to 5% (v/v) and 25% (v/v). Absorbance spectra (400–800 nm) were monitored via a UV–vis spectrometer.

## Supplementary Material



## Notes

Histogram of the length distribution of CuNW, TEM images after the ligand-exchange process, characterization and kinetic analysis of Cu_2_O nanoparticles, results of the salt study, comparisons of absorbance vs time for Cu-based metallic nanostructures and Au nanoparticles, and tables for *k*_cat_ calculated using substrate concentrations under different conditions, as well as comparisons of kinetic parameters with literature values (PDF)
