# Supplementary material for: Copper-Based Nanostructures as Peroxidase Mimics: Elucidating the Roles of Shape, Composition, and Chloride Ion Modulation
Source: ACS Nanosci Au. 2026 Oct 13;6(5):1037–49. doi: 10.1021/acsnanoscienceau.6c00146 (PMC13693252; doi:10.1021/acsnanoscienceau.6c00146)
Supplement: Supplementary file 1 [file ng6c00146_si_001.pdf]

Supporting Information

Copper-Based Nanostructures as Peroxidase Mimics: Elucidating the Roles of Shape, Composition, and Chloride Ion Modulation

Ghazaleh Salmanian,[1,2] Lihua Zhang,[3] Mourad Benamara,[4] Abigail Bell,[1] Jingyi Chen[1,2,*]

[1]*Department of Chemistry and Biochemistry, University of Arkansas, Fayetteville, AR 72701, United States*

[2]*Materials Science and Engineering Graduate Program, University of Arkansas, Fayetteville, AR 72701, United States*

[3]*Center for Functional Nanomaterials, Brookhaven National Laboratory, Upton, NY 11973, United States*

[4]*Arkansas Nano & Bio Materials Characterization Facility, University of Arkansas, Fayetteville, AR 72701, United states*

[*]Email: chenj@uark.edu

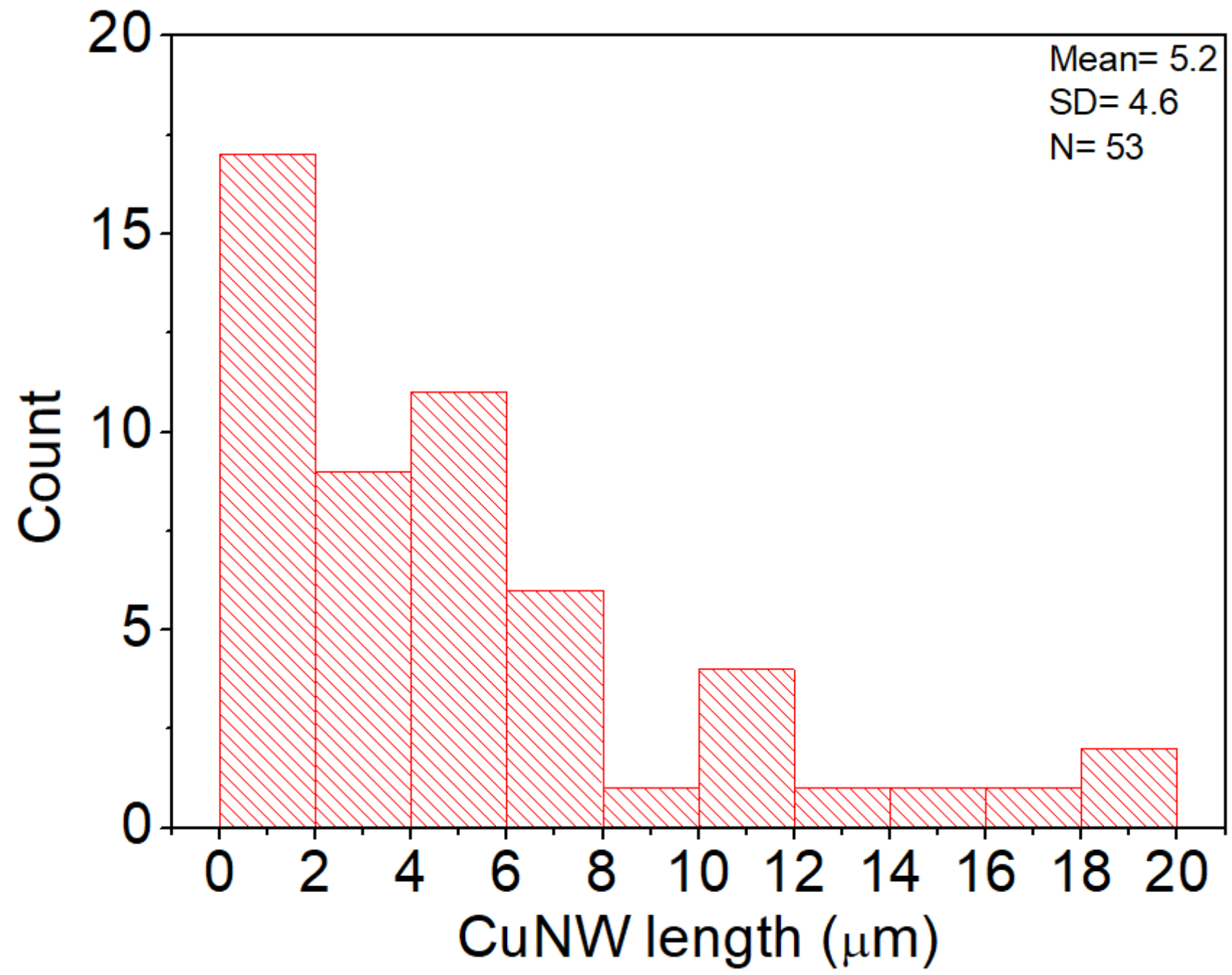

Figure S1. Histogram of Cu nanowire (CuNW) length measurement. The measured CuNW lengths have a mean of 5.2 μm and a standard deviation of 4.6 μm (n=53).

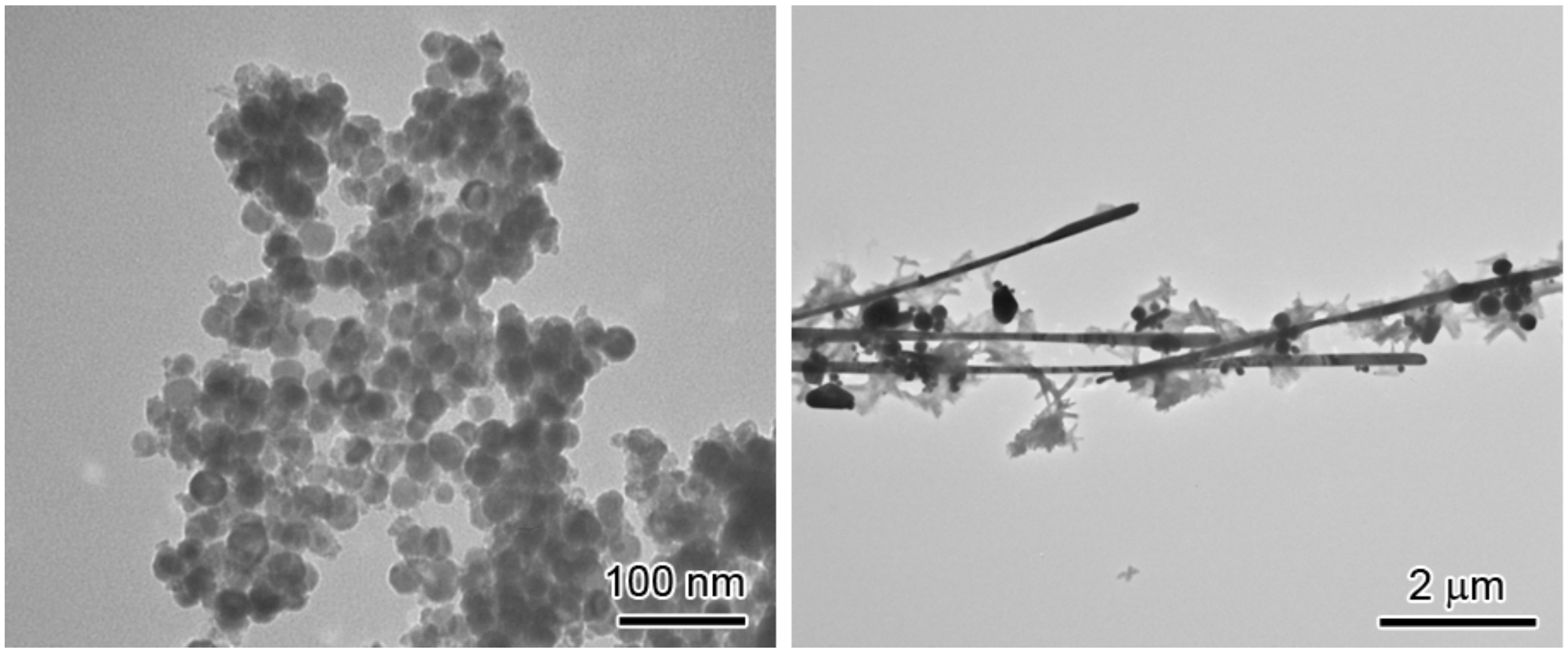

Figure S2. TEM images of the Cu nanostructures after a ligand exchange process: (A) spherical Cu nanoparticles (Cu nanospheres, CuNS); and (B) TEM image of Cu nanowires (CuNW), indicating the nanostructures remained unchanged.

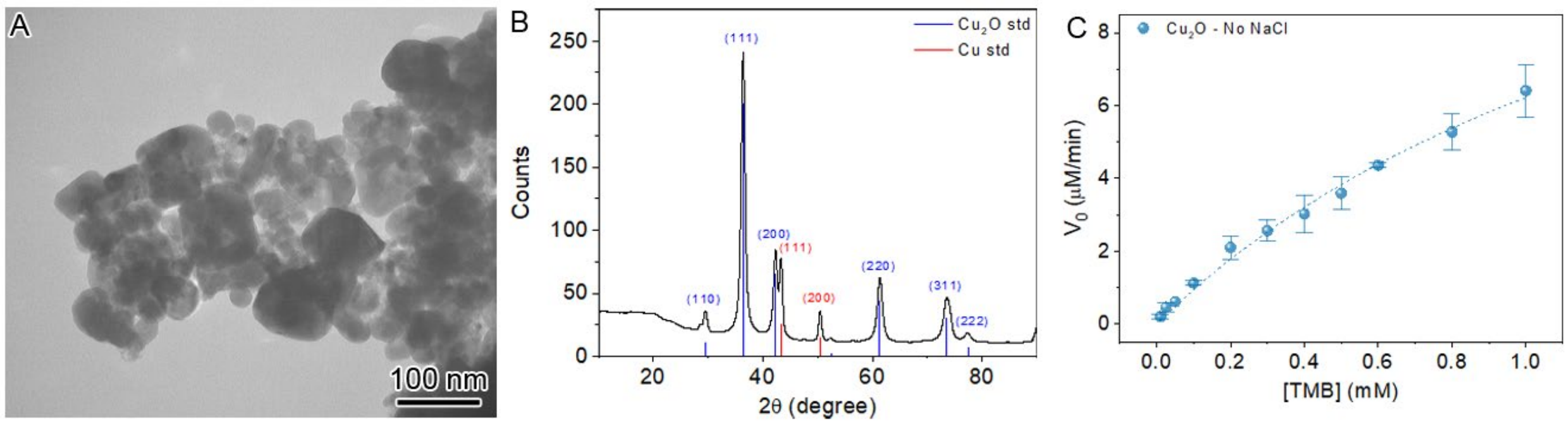

Figure S3. Characterization and kinetic measurements of Cu_2O nanoparticles obtained from aging CuNS in an oxygenated environment: (A) TEM image; (B) XRD pattern indicating that it consists mostly of Cu_2O with some Cu remaining; (C) Michaelis-Menten plot of TMB oxidation on Cu_2O.

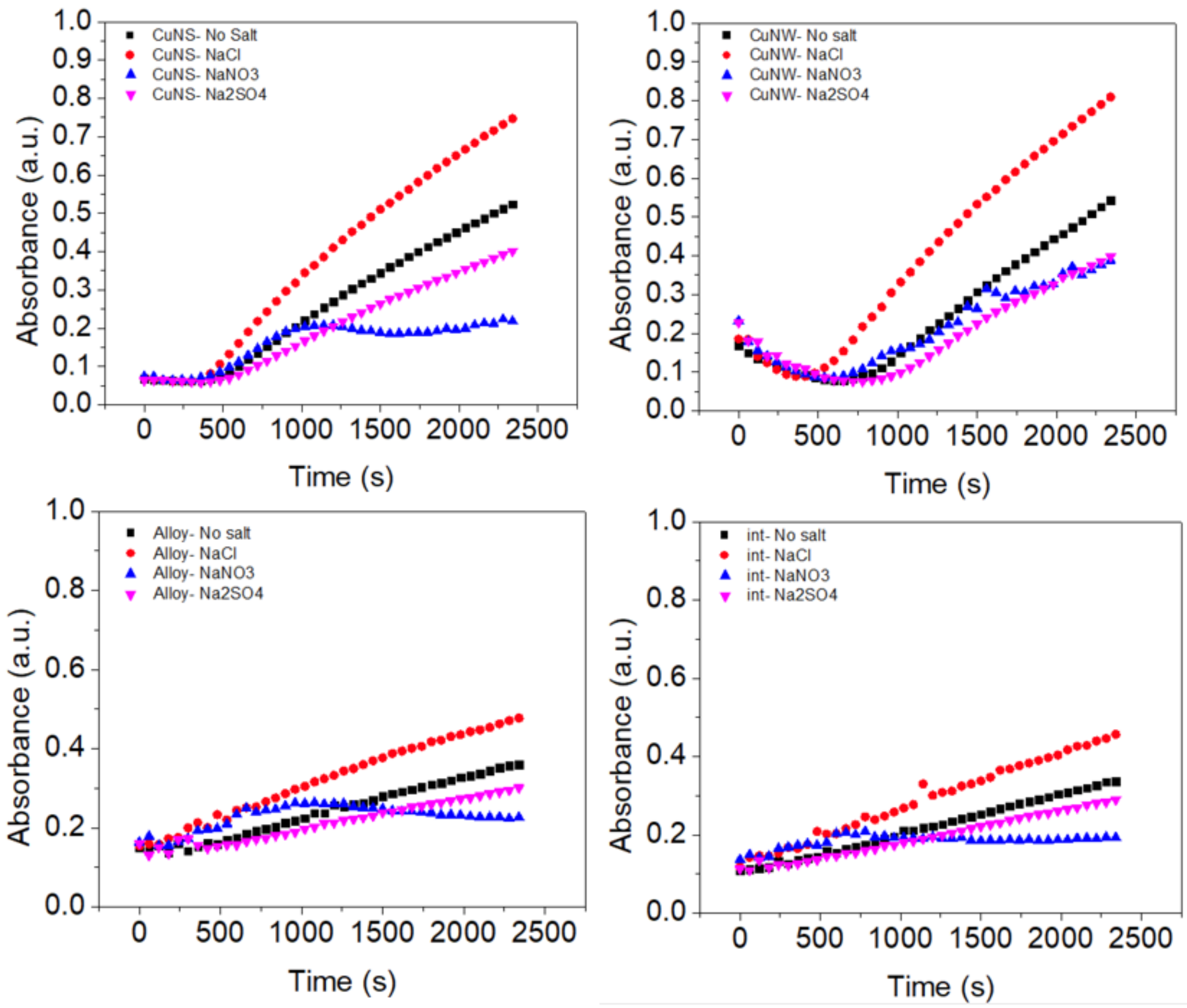

Figure S4A. Time-dependent absorbance of Cu nanospheres (CuNS), Cu nanowires (CuNW), Cu_3Au alloy, and Cu_3Au intermetallic (int) in the absence of salt (No salt) and in the presence of NaCl, $NaNO_3$, and Na_2SO_4. Absorbance was monitored as a function of reaction time at 652 nm to evaluate the effect of different anions on the catalytic activity of the Cu-based nanostructures.

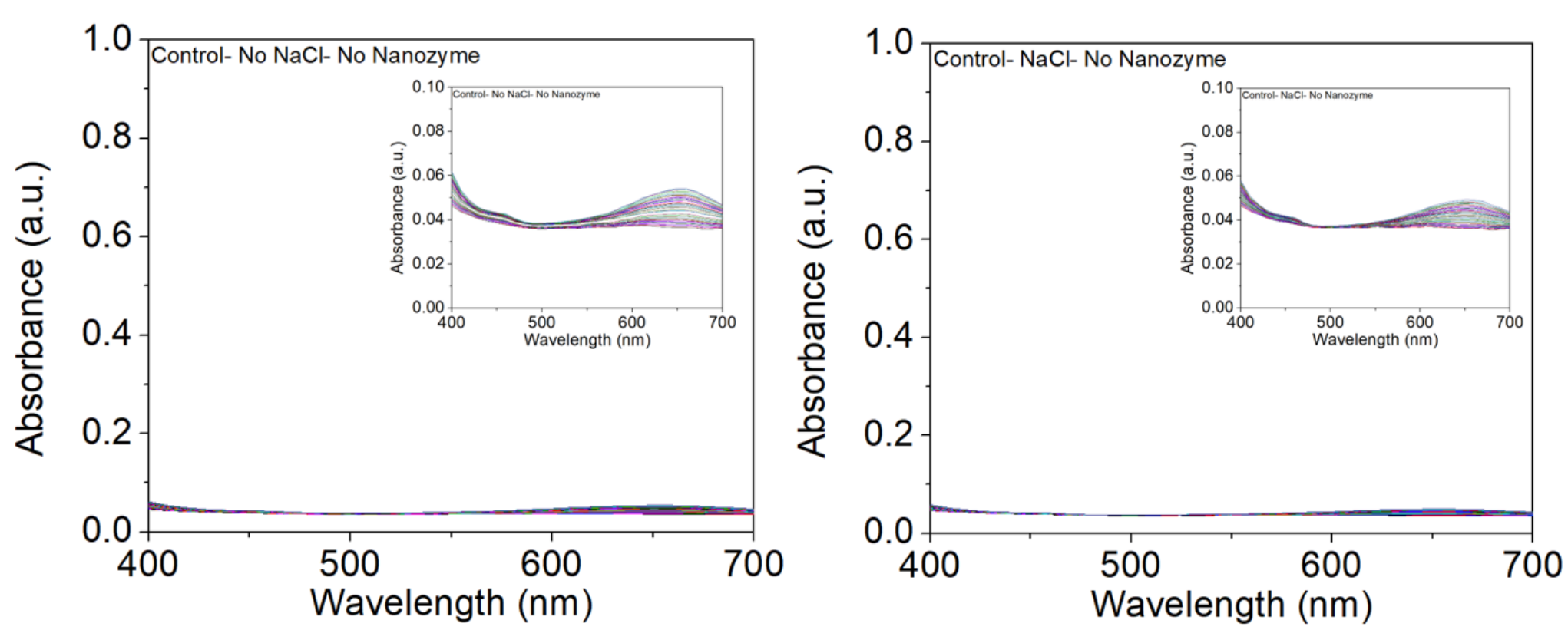

Figure S4B. Time-dependent UV-Vis spectra of TMB and H_2O_2 in the absence of nanozyme, recorded over 40 minutes, (left) without NaCl and (right) with NaCl. Insets show the 0-0.1 absorbance range.

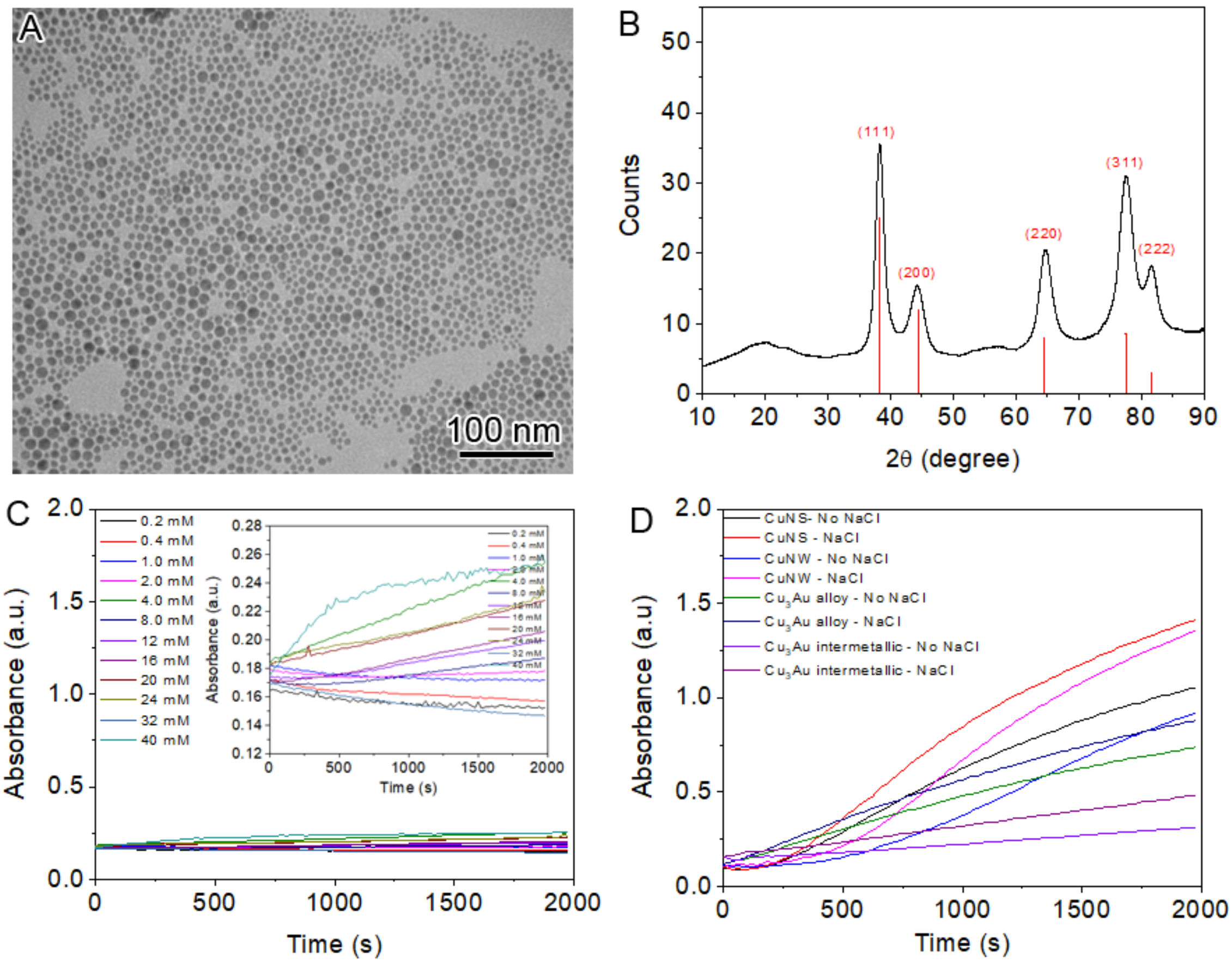

Figure S5. (A-C) Characterization and kinetic measurements of Au nanoparticles: (A) TEM image; (B) XRD pattern that has been indexed according to the Au standard from Materials Project (MP-81) labeled in red; (C) Plots of absorbance vs. time of TMB oxidation at varying TMB concentrations, indicating that the catalytic activity is low, with a zoom-in inset. The labeled stock TMB concentration was diluted by taking a 50 µL aliquot and diluting it to 2 mL. (D) Comparison of the plots of absorbance vs. time of TMB oxidation on different Cu-based metallic nanostructures in the absence and presence of NaCl under the same conditions: 10 µg/mL particles, 0.4 µmol TMB, 40 µmol H_2O_2 in 2 mL.

Table S1A. Apparent k_{cat} and k_{cat}/K_m normalized per nanoparticles. Each nanoparticle was treated as a single catalytic entity, giving nanoparticle molar concentration of 2.92×10^{-10} M (CuNS), 2.26×10^{-13} M (CuNW), and 1.04×10^{-10} M (Cu_2O/Cu NP) at a loading of 0.02 mg in 2 mL.

Nanozyme	Substrate	k_{cat} (s^{-1})		k_{cat}/K_m (μM^{-1} s^{-1})	
		No NaCl	NaCl	No NaCl	NaCl
CuNS	TMB	254.00	592.46	0.70	1.08
	H_2O_2	621. 57	850. 45	0.0052	0.01
CuNW	TMB	697640.12	1370206.49	459.00	1132.40
	H_2O_2	667404.13	856932.15	6.21	17.63
Cu_2O NS	TMB	1567.78	-	1.60	-

Table S1B. Apparent k_{cat} and k_{cat}/K_m normalized per surface atom. Every estimated surface Cu atom was assumed to be an active site, giving surface atom concentrations of 7.7×10^{-6} M (CuNS), 2.71×10^{-6} M (CuNW), and 5.50×10^{-6} M (Cu_2O/Cu NP) at a loading of 0.02 mg in 2 mL.

Nanozyme	Substrate	k_{cat} ($\times 10^{-4}$ s^{-1})		k_{cat}/K_m ($\times 10^{-6}$ μM^{-1} s^{-1})	
		No NaCl	NaCl	No NaCl	NaCl
CuNS	TMB	96.32	224.67	26.75	40.85
	H_2O_2	235.71	322.51	0.20	0.40
CuNW	TMB	581.80	1142.70	38.27	94.43
	H_2O_2	556.58	714.64	0.52	1.47
Cu_2O NS	TMB	296.45		38.50	

Table S1C. Mass normalized apparent k_{cat} and k_{cat}/K_m. V_{max} was normalized to the Cu mass concentration in the assay (10 mg/L; 0.02 mg in 2 mL). This normalization makes no assumption about active site number or accessibility.

Nanozyme	Substrate	k_{cat} (nmol/mg.s)		k_{cat}/K_m (nmol/mg.mM.s)	
		No NaCl	NaCl	No NaCl	NaCl
CuNS	TMB	7.42	17.30	20.00	31.50
	H_2O_2	18.15	24.83	0.15	0.30
CuNW	TMB	15.76	30.97	10.40	25.60
	H_2O_2	15.08	19.37	0.14	0.40
Cu_2O NS	TMB	16.31	-	21.20	-

Table S1D. Apparent k_{cat} and k_{cat}/K_m normalized per nanoparticles. Each nanoparticle was treated as a single catalytic entity, giving nanoparticle molar concentration of 1.92×10^{-9} M (CuNS) and 3.01×10^{-9} M (CuNW) at a loading of 0.02 mg in 2 mL.

Nanozyme	Substrate	k_{cat} (s^{-1})		k_{cat}/K_m ($\mu M^{-1} s^{-1}$)	
		No NaCl	NaCl	No NaCl	NaCl
Cu_3Au	TMB	19.79	99.83	0.032	0.041
alloy	H_2O_2	40.54	70.83	0.00058	0.0006
Cu_3Au	TMB	6.97	21.26	0.004	0.0097
intermetallic	H_2O_2	8.14	7. 20	5.5E-5	9.06E-5

Table S1E. Apparent k_{cat} and k_{cat}/K_m normalized per surface atom. Every estimated surface atom (Cu+Au) was assumed to be an active site, giving surface-atom concentration of 1.32 $\times 10^{-5}$ M (alloy) and 1.51 $\times 10^{-5}$ M (intermetallic).

Nanozyme	Substrate	k_{cat} ($\times 10^{-4}$ s^{-1})		k_{cat}/K_m ($\times 10^{-6}$ μM^{-1} s^{-1})	
		No NaCl	NaCl	No NaCl	NaCl
Cu_3Au	TMB	28.78	145.20	4.72	5.97
alloy	H_2O_2	58.96	103.03	0.08	0.087
Cu_3Au	TMB	13.90	42.38	0.88	1.93
intermetallic	H_2O_2	16.22	14.34	0.01	0.02

Table S1F. Cu mass-normalized apparent k_{cat} and k_{cat}/K_m. V_{max} was normalized to the Cu mass concentration in the assay (7.5 mg/L Cu), calculated from the ICP-MS Cu:Au mass ration of 3:1.

Nanozyme	Substrate	k_{cat} (nmol/mg.s)		k_{cat}/K_m (nmol/mg.mM.s)	
		No NaCl	NaCl	No NaCl	NaCl
Cu_3Au	TMB	5.07	25.55	8.31	10.51
alloy	H_2O_2	10.38	18.13	0.15	0.15
Cu_3Au	TMB	2.80	8.53	1.77	3.90
intermetallic	H_2O_2	3.27	2.88	0.02	0.04

Table S2. Results from representative literature references.

	[E] (M)	Substrate	K_m (mM)	V_{max} (M.S^{-1})	K_{cat} (S^{-1})	Ref
**Au NPs**	7.64 x10^{-9} Mass	TMB	0.20	5.59 x 10^{-8}	734.38	1
	7.64 x10^{-9} Mass	H_2O_2	572.98	9.77 x 10^{-8}	1,284	
**CuO NPs**	-	TMB	0.013	-	-	2
	-	H_2O_2	85.6	-	-	
**CuO-Au alloy**	-	TMB	3.54	0.0111	-	3
	-	H_2O_2	4.08	0.0105	-	
**CuO**	-	TMB	25	10.49 x 10^{-8}	-	4
		H_2O_2	400	16.1 x 10^{-8}	-	
**Fe_3O_4**	11.4 x 10^{-13}	TMB	0.098	3.44 x 10^{-8}	3.02 x 10^4	5
	11.4 x 10^{-13}	H_2O_2	154	9.78 x 10^{-8}	8.58 x 10^4	
**CuO**	-	TMB	0.016	-	-	6
		H_2O_2	41.0	-	-	
**Au NPs**		TMB	25.21	15.57 x 10^{-6}	5990.9	7
		H_2O_2	-	-	-	

References:

(1) Lou, D.; Tian, Y.; Zhang, Y.; Yin, J.; Yang, T.; He, C.; Ma, M.; Yu, W.; Gu, N. Peroxidase-Like Activity of Gold Nanoparticles and Their Gold Staining Enhanced ELISA Application. *J. Nanosci. Nanotechnol.* **2018**, *18* (2), 951–958. DOI: 10.1166/jnn.2018.13977.

(2) Chen, W.; Chen, J.; Liu, A.-L.; Wang, L.-M.; Li, G.-W.; Lin, X.-H. Peroxidase-Like Activity of Cupric Oxide Nanoparticle. *ChemCatChem* **2011**, *3* (7), 1151–1154. DOI: 10.1002/cctc.201100064.

(3) Mvango, S.; Mashazi, P. Synthesis, Characterization of Copper Oxide-Gold Nanoalloys and Their Peroxidase-Like Activity Towards Colorimetric Detection of Hydrogen Peroxide and Glucose. *Mater. Sci. Eng., C* **2019**, *96*, 814–823. DOI: 10.1016/j.msec.2018.12.010.

(4) Nagvenkar, A. P.; Gedanken, A. $Cu_{0.89}Zn_{0.11}O$, A New Peroxidase-Mimicking Nanozyme with High Sensitivity for Glucose and Antioxidant Detection. *ACS Appl. Mater. Interfaces* **2016**, *8* (34), 22301–22308. DOI: 10.1021/acsami.6b05354.

(5) Gao, L.; Zhuang, J.; Nie, L.; Zhang, J.; Zhang, Y.; Gu, N.; Wang, T.; Feng, J.; Yang, D.; Perrett, S.; Yan, X. Intrinsic Peroxidase-Like Activity of Ferromagnetic Nanoparticles. *Nat. Nanotechnol.* **2007**, *2* (9), 577–583. DOI: 10.1038/nnano.2007.260.

(6) Chen, W.; Chen, J.; Feng, Y.-B.; Hong, L.; Chen, Q.-Y.; Wu, L.-F.; Lin, X.-H.; Xia, X.-H. Peroxidase-Like Activity of Water-Soluble Cupric Oxide Nanoparticles and Its Analytical Application for Detection of Hydrogen Peroxide and Glucose. *Analyst* **2012**, *137* (7), 1706–1712. DOI: 10.1039/c2an35072f.

(7) Wang, C.; Shi, Y.; Dan, Y.-Y.; Nie, X.-G.; Li, J.; Xia, X.-H. Enhanced Peroxidase-Like Performance of Gold Nanoparticles by Hot Electrons. *Chem. - Eur. J.* **2017**, *23* (28), 6717–6723. DOI: 10.1002/chem.201605380.
